# Cu(i)-catalysed 1,2,3-triazole stitched chalcomer assembly as Pb(ii) and Cu(ii) ion sensor: DFT and docking scrutiny†

**DOI:** 10.1039/d3ra05760g

**Published:** 2023-11-03

**Authors:** Riddima Singh, Gurleen Singh, Nancy George, Gurjaspreet Singh, Anita Devi, Harminder Singh, Gurpreet Kaur, Jandeep Singh

**Affiliations:** a School of Chemical Engineering and Physical Sciences, Lovely Professional University Phagwara-144411 Punjab India singhjandeep@gmail.com; b Department of Chemistry and Centre of Advanced Studies in Chemistry, Panjab University Chandigarh-160014 Punjab India; c Department of Chemistry, Gujranwala Guru Nanak Khalsa College Civil Lines Ludhiana-141001 Punjab India

## Abstract

Herein, a 1,2,3-triazole derivative (CBT), synthesized using the Copper(i) catalyzed Alkyne Azide Cycloaddition (CuAAC) procedure, based on a chalcone skeleton has been reported, that was implemented as an effective sensor for Pb(ii) and Cu(ii) ions. The synthesized CBT was characterized using spectroscopic techniques such as FTIR, NMR (^1^H and ^13^C), and mass spectrometry. The sensing behaviour of CBT was analyzed using UV-Vis spectroscopy, demonstrating selective sensing for Pb(ii) and Cu(ii) ions, competitively. The correlation plot revealed the detection limit for Pb(ii) and Cu(ii) ions to be 100 μM and 110 μM respectively. In addition, DFT simulations and molecular electrostatic potential (MEP) studies scrutinized the binding strategy of the free CBT and its orientation towards the metal ions in the metal–ligand complex. The probe CBT was predicted *via* the online platform Way2drug for its pharmacological properties, investigating the possibility to inhibit early atherosclerosis. CBT was subsequently docked to the TRIB1 protein using AutoDock Vina and demonstrated a high binding affinity with a value of −6.2 kcal mol^−1^.

## Abstract 2

1,2,3-triazole derivative (CBT) was synthesized using the CuAAC procedure, with chalcone skeleton and was implemented as an effective sensor for Pb(ii) and Cu(ii) ions.

## Graphical abstract

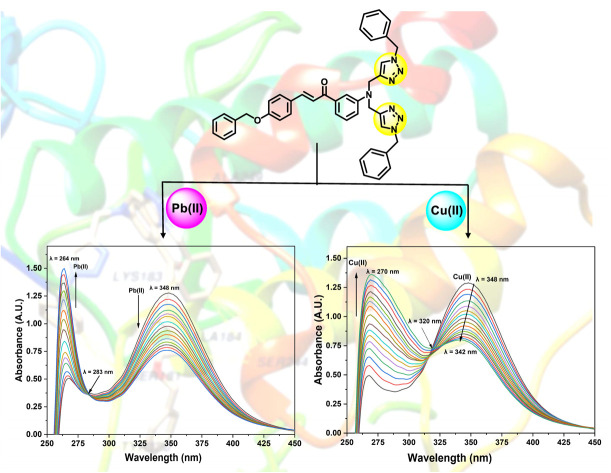

## 1. Introduction

The environmental accumulation of metal ions above the permissible concentrations has led to their substantial upsurge, which has put forward the need to explore efficient and robust research alternatives for their instantaneous detection even in low concentrations. The emphasis on analytical cation detection is due to their well-documented pervasive toxic effects on both the terrestrial as well as aquatic living systems,^1–3^ thereby rendering their qualitative as well as quantitative recognition as the need of the hour.^4^ Due to the unregulated use of lead *via* mining, smelting, ceramics, paint, automobile exhaust emissions, *etc.*, its accumulation in the environment has undergone an exponential ascent since the latter years of the ‘bygone century’.^5^ Also, exposure to lead can denature DNA and proteins, is detrimental to bone health, and may result in cell transformation, thereby rendering the cells to become malignant.^6^ Copper ions, though involved in biological processes in electron and oxygen transport in the body's soft tissues, can be toxic in higher concentrations where they can interfere with biological functioning by altering essential proteins/enzymes.^7–9^ This can pose serious health risks like anaemia, interstitial nephritis, oxidative damage, hypertension, cardiovascular disease, severe neurological disease, *etc.*^10,11^ Therefore, the detection of these metal ions above the threshold limit needs immediate attention to address environmental pollution and minimize impacts on biological systems.^12,13^ This leads to the importance of metal ion sensors based on organic frameworks for selective recognition.

In this pursuit, *O*-chalcones and N-heterocycles have been potential sensors due to their selective cation binding capabilities.^14^ The chalcones have a backbone of unsaturated carbonyl groups with conjugation and have the potential to exhibit chemosensing.^15–17^ The combination of these motifs with the 1,2,3-triazole moiety *via* ‘Click’ (CuAAC) methodology has emerged as the most preferred synthetic route to form chalcone appended 1,4-disubstituted-1,2,3-triazole derivatives.^18^ There is an increased demand for these moieties owing to their extensive selectivity for ion detection,^19,20^ in addition to their significant biological properties such as antibacterial, antioxidative, antifungal activities *etc.*

The ion detecting property of a fluorescent chemosensor is based on a host–guest relationship, wherein the interaction of the receptor with the analyte induces photophysical changes in the fluorophore which indicates successful ion recognition.^21^ Besides, exclusivity of a sensor is attributable to the cavity size, wherein the N-rich 1,2,3-triazole ring helps in the recognition process by behaving as a linker between the fluorophore and the receptor unit.^5^ Furthermore, the chemosensor must have a lower limit of detection (LOD) and limit of quantification (LOQ) for efficient ion sensing.^22,23^

The 2022 Nobel Prize winning ‘Click Chemistry’ reaction was exploited for the synthesis of a chalcone-based 1,2,3-triazole derivative (CBT), that was a selective Pb(ii) and Cu(ii) ion sensor, as explored *via* UV-vis spectroscopy. The Density Functional Theory (DFT) for the CBT and its corresponding metal complex was calculated using the (B3LYP)/631G+(d,p) basis set of theory and LANL2DZ basis set for the CBT-metal complex. Using the energy-optimized structure, the CBT was subsequently docked with TRIB1 protein to analyze its anti-atherosclerosis properties. While several 1,2,3-triazole-based sensors for Pb(ii) as well as Cu(ii) have been reported in the past,^24^ this is the first study, as per our knowledge demonstrating that a single chalcone-based 1,2,3-triazole-appended ligand serves as a suitable detection agent for the simultaneous sensing of Pb(ii)and Cu(ii) ions.

## 2. Synthesis

**Alert!** Sodium azide is shock & heat sensitive and must be handled cautiously.

### 2.1. Materials and method

The materials used in this reported synthesis were 4-benzyloxybenzaldehyde (Spectrochem), 3-amino acetophenone (LOBA), potassium hydroxide (CDH), ethyl acetate (LOBA), hexane (LOBA), anhydrous potassium carbonate (LOBA), propargyl bromide (80% by weight in toluene) (Spectrochem), [CuBr(PPh_3_)_3_] (Sigma Aldrich), tetrahydrofuran (THF) (LOBA), ethanol, benzyl chloride (LOBA), triethylamine (Et_3_N) (SDFCL), sodium azide (LOBA), *N*,*N*-dimethylformamide (DMF) (LOBA). The chloride salts of Ba(ii), Ca(ii), Co(ii), Cr(iii), Cu(ii), Hg(ii), Mg(ii), Ni(ii), Pb(ii), and Zn(ii), Ce(iii), Mn(ii), Cd(ii), Na(i), K(i) were bought from LOBA. Benzyl azide was synthesized by reacting benzyl chloride with dried sodium azide by a known procedure.^23^ The spectroscopic analysis was carried out using SHIMADZU FTIR-8400S Spectrometer and BRUKER-ADVANCE-II FT-NMR-AL 500 MHz spectrometer. The chemical shifts in NMR were recorded against tetramethylsilane considered as a standard reference. Mass spectrometry (LCMS) was performed on Bruker make mass spectrometer model Esquire 300 mass spectrometer and melting point was detected with Labtronics LT 108 device. The PerkinElmer Model 2400 CHNS elemental analyzer was used to obtain CHN analyses and for chemosensing analysis, SHIMADZU UV-1900 Spectrometer was used. For DFT analysis, Gaussian 09 software with hybrid density functional (B3LYP)/631G+(d,p) basis set and LANL2DZ basis set was employed, and AutoDock Vina was used for molecular docking.

### 2.2. Synthesis of CBT

**Scheme 1:**
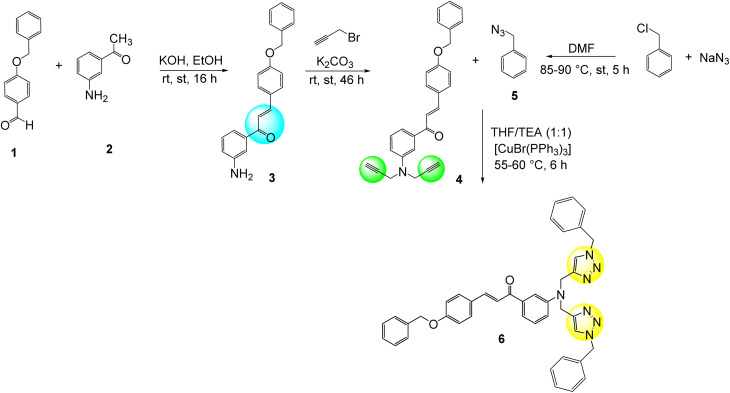
Schematic representation for the synthesis of chalcone-based 1,2,3-triazole (CBT).

The synthesis of chalcone-based 1,2,3-triazole (CBT) was carried out in a three-step sequential pathway (Scheme 1) wherein initially the chalcone (3) was synthesized from 4-benzyloxybenzaldehyde (1) and 3-aminoacetophenone (2) as described in sub-Section 2.2.1. The chalcone (3) was subsequently converted into its corresponding terminal alkyne (4) by reaction with propargyl bromide (sub-Section 2.2.2.) and in the final step, the terminal alkyne (4) was reacted with benzyl azide (5) to synthesize the corresponding 1,2,3-triazole derivative (6) (sub-Section 2.2.4).

#### 2.2.1. Synthesis of chalcone (3)

4-Benzyloxybenzaldehyde (2.0 g, 9.4 mmol) (1) and 3-aminoacetophenone (1.25 g, 9.4 mmol) (2) were dissolved in ethanol with continuous stirring. To the reaction mixture, potassium hydroxide (5.0 mL, 20% w/v) was added slowly. The reaction mixture was continuously stirred at room temperature until the complete conversion of reactants into the desired product (3), while the reaction progress was supervised by TLC (ethyl acetate : hexane; 1 : 9). The reaction was quenched by the addition of ice-cold water into the reaction mixture, and the solid product was then filtered and dried. The product obtained was then purified using ethanol as an eluent.^25^

##### 2.2.1.1. (*E*)-1-(3-Aminophenyl)-3-(4-(benzyloxy)phenyl)prop-2-en-1-one (3)

Yield: 87%; colour/texture: light yellow powder; M.F. = C_22_H_19_NO_2_; elem. anal. calc. (%): C = 80.22; H = 5.81; N = 4.25; found (%): C = 80.29, H = 5.89, N = 4.31; mp: 121–122 °C; IR (neat, cm^−1^): 3447, 3357 (–NH_2_, % *T* = 66), 3061 (aromatic C–H str., % *T* = 77), 2892 (aliphatic C–H str., % *T* = 79), 1654 (C=O, % *T* = 58), 1627 (C=C, % *T* = 68), 1570, 1507 (aromatic C=C, % *T* = 36), 1453 (–CH_2_ bend, % *T* = 46), 1381, 1334, 1287, 1248, 1168, 1115, 1035, 1015.

#### 2.2.2. Synthesis of the chalcone-based alkyne (4)

Chalcone (3) (1.0 g, 3.03 mmol) was dissolved in 20 mL DMF by swirling the mixture constantly on the magnetic stirrer. To this solution, anhydrous potassium carbonate (2.1 g, 15.2 mmol) was added, immediately followed by the addition of propargyl bromide (0.78 g, 6.88 mmol) in a dropwise manner. The reaction was stirred at room temperature for 46 h. The completion of the reaction was monitored by TLC (ethyl acetate : hexane; 1 : 9). Thereafter, ice-cold water was used for quenching the reaction mixture, the solid product so obtained was filtered and dried at room temperature.

##### 2.2.2.1. (*E*)-3-(4-(Benzyloxy)phenyl)-1-(3-(di(prop-2-yn-1-yl)amino)phenyl)prop-2-en-1-one (4)

Yield: 81%; colour/texture: light brown powder; M.F. = C_28_H_23_NO_2_; elem. anal. calc. (%): C = 82.94; H = 5.72; N = 3.45; found (%): C = 82.10; H = 5.81; N = 3.54; mp: 112–113 °C; IR (neat, cm^−1^): 3279 (C≡*C–H* str., % *T* = 78), 3066, 3033 (aromatic C–H str., % *T* = 88, 87), 2918 (aliphatic C–H str., % *T* = 87), 2111 (C≡C str., % *T* = 89), 1655 (C=O str., % *T* = 76), 1570, 1505 (aromatic C=C str., % *T* = 60,67), 1451 (–CH_2_ bend, % *T* = 71), 1381, 1290 (C–N str., % *T* = 76,71), 1237, 1167 (C–O str., % *T* = 56, 59), 1115, 1073; ^1^H NMR (500 MHz, CDCl_3_): *δ* = 7.76 (d, *J* = 15.6 Hz, 1H), 7.57 (d, *J* = 8.2 Hz, 2H), 7.49 (d, *J* = 7.4 Hz, 1H), 7.40 (dd, *J* = 24.5, 10.3 Hz, 9H), 6.99 (d, *J* = 8.4 Hz, 2H), 5.09 (s, 2H), 4.17 (s, 4H), 2.27 (s, 2H) ppm; ^13^C NMR (126 MHz, CDCl_3_): *δ* = 190.81, 160.82, 147.96, 144.55, 136.45, 130.23, 129.31, 128.69, 128.19, 127.96, 127.49, 120.29, 119.87, 119.71, 115.33, 115.17, 78.87, 73.02, 70.14, 40.53 ppm.

#### 2.2.3. General procedure for the synthesis of benzyl azide (5)

Benzyl chloride (5.5 g, 47.8 mmol) was dissolved in DMF (25 mL) using a magnetic stirrer, followed by the addition of sodium azide (15.5 g, 239 mmol). The reaction mixture was heated to 85–90 °C, while allowing it to reflux for 4–5 hours. TLC analysis confirmed the successful completion of the reaction (ethyl acetate : hexane; 1 : 4). Ethyl acetate was used in the solvent extraction process to obtain the product. The combined organic layers were separated and dried with anhydrous sodium sulphate, filtered, and evaporated in a vacuum to eliminate any leftover solvent.

Yield: 61%; color/texture: light yellow oil; M.F.: C_7_H_7_N_3_; IR (neat, cm^−1^): 3032 (aromatic C–H str.), 2930 (aliphatic C–H str.), 2089 (–N_3_ str., % *T* = 40), 1452 (–CH_2_ bend, % *T* = 95), 1252 (C–N str., % *T* = 70), 876, 697 (monosubstituted ring, % *T* = 95, 65), 568; ^1^H NMR (500 MHz, CDCl_3_) *δ* = 7.27–7.12 (m, 5H), 4.14 (s, 2H) ppm; ^13^C NMR (126 MHz, CDCl_3_) *δ* = 135.53, 128.91, 128.37, 128.31, 54.82 ppm.

#### 2.2.4. Synthesis and characterization of chalcone-based triazole derivative (6) (CBT)

To the solution of chalcone-based alkyne 4 (0.70 g, 1.73 mmol) in the THF : TEA (3 : 2), the organic azide (0.46 g, 3.46 mmol), and the Cu(i) catalyst (0.001 mmol) was added. The reaction mixture was refluxed for 5 hours at 55–60 °C until the complete conversion of reactants into the desired product and was observed by TLC (ethyl acetate : hexane; 1 : 4). The quenching of the reaction was done by the addition of ice-cold water into the reaction mixture, and the solid product was then filtered and dried.

##### 2.2.4.1. (*E*)-3-(4-(Benzyloxy)phenyl)-1-(3-(bis((1-benzyl-1*H*-1,2,3-triazol-4 yl)methyl)amino)phenyl)prop-2-en-1-one (6) (CBT)

Yield: 79%; colour/texture: light brown powder; M.F.: C_42_H_37_N_7_O_2_; elem. anal. calc. (%): C = 75.09; H = 5.55; N = 14.59, found (%): C = 75.15; H = 5.47; N = 14.52, mp: 162–163 °C; IR (neat, cm^−1^): 3120, 3069, 3030 (aromatic C–H str., % *T* = 93, 92, 92), 2903 (aliphatic C–H str., % *T* = 93), 1653 (C=O str., % *T* = 87), 1570, 1511, 1492 (aromatic C=C str., % *T* = 75, 83, 84), 1450 (–CH_2_ bend, % *T* = 81), 1330, 1291 (C–N str., % *T* = 84, 86), 1250, 1174 (C–O str., % *T* = 77), 1121, 1028; ^1^H NMR (500 MHz, DMSO-d6): *δ* = 8.03 (s, 2H), 7.83–7.82 (d, 2H), 7.36–7.67 (s, 2H), 7.56 (s, 1H), 7.48–7.39 (m, 6H), 7.35–7.28 (m, 7H), 7.23–7.22 (m, 4H), 7.17–7.08 (m, 3H), 5.54 (s, 4H), 5.19 (s, 2H), 4.73 (s, 4H) ppm; ^13^C NMR (126 MHz, DMSO-d6): 189.31, 160.27, 147.82, 144.48, 143.34, 138.55, 136.60, 136, 130.55, 129.12, 128.57, 128.37, 127.90, 127.84, 127.65, 127.58, 127.45, 123.23, 119.92, 117.28, 116.72, 115.15, 112.36, 69.29, 52.61, 45.75 ppm; LC-MS: *m*/*z* (calculated): 671.81, *m*/*z* (observed): 672.30 (M + 1).

## 3. Result and discussion

### 3.1. Synthesis

The synthesis of CBT probe follows three-step procedure wherein initial step is chalcone synthesis, followed by the synthesis of alkyne from the priorly formed chalcone through nucleophilic substitution with propargyl group using anhydrous potassium carbonate as a base. As a result, the labile protons of the chalcone were replaced with propargyl groups yielding terminal alkyne as a product. Subsequently, in the final step, azide was fused with the alkyne in the presence of THF as a solvent, triethylamine (TEA) as a base, and [CuBr(PPh_3_)_3_] as a catalyst to achieve the final product. The final step follows the principle of Green Chemistry where in complete atom economy was observed.

### 3.2. Spectroscopic analysis

#### 3.2.1. IR spectroscopy

The appearance of sharp signal at 1627 cm^−1^ corresponding to the formation of C=C in the IR spectrum, confirmed synthesis of chalcone, since this peak was absent in the IR spectra of either of the reactants. The peaks at 3447 cm^−1^ and 3357 cm^−1^ corresponded to the *N*–H stretching of the NH_2_ group present in the chalcone. In the IR spectrum of alkyne 4, the peaks at 3279 cm^−1^ and 2111 cm^−1^ corresponded to the –C≡*C–H* and –*C*≡*C*– stretching, respectively, thereby confirming the successful formation of the alkyne. The sharp peak of high intensity at 2089 cm^−1^ in the IR spectrum of benzyl azide due to the –N_3_ group confirmed its successful formation. In the IR spectrum of CBT, the disappearance of peaks at 3279 cm^−1^ due to –C≡*C–H* stretching, 2111 cm^−1^ due to –*C*≡*C*– unit and 2089 cm^−1^ due to the –N_3_ group of benzyl azide indicated the merging of –C≡C–H functional group of alkyne with the azide to form the 1,2,3-triazole ring. In addition to this, the peak at 3120 cm^−1^ due to C–H stretching of the 1,2,3-triazole moiety, 3069 cm^−1^ due to aromatic C–H stretching, and 2903 cm^−1^ due to aliphatic C–H stretching confirms the successful formation of the desired product.

#### 3.2.2. NMR spectroscopy and mass spectrometry

^1^H and ^13^C NMR spectra of the alkyne 4 and CBT confirmed the synthesis of both compounds. The peak at *δ* = 2.27 ppm in the ^1^H NMR spectrum of alkyne 4 corresponded to the alkynyl proton, which was missing in the ^1^H NMR spectrum of CBT, confirming the successful conversion of alkynyl group to produce the 1,2,3-triazole moiety during the cycloaddition process. Moreover, the peak at *δ* = 4.17 ppm due to –CH_2_–*N*– protons in the ^1^H NMR spectrum of alkyne were displaced downfield, as observed at *δ* = 4.58 ppm in the ^1^H NMR spectrum of CBT. In addition to this, the protons attached to the 1,2,3-triazole ring emerged as sharp peak at *δ* = 7.67 ppm in the ^1^H NMR spectrum of CBT. In the ^13^C NMR spectrum of alkyne 4, the peaks belonging to the C≡C moiety were seen at *δ* = 73.02 ppm and *δ* = 78.87 ppm. However, these peaks were absent in the ^13^C NMR spectrum of CBT, thereby confirming the successful conversion of the alkyne moiety into the 1,2,3-triazole ring. To confirm the formation of pure product, the mass spectrum was also analysed, and a peak observed at *m*/*z* = 672.30 evidenced the successful synthesis of CBT and spectroscopic data is provided in the ESI.†

### 3.3. UV-vis studies to analyze the chemosensing behavior

**Fig. 1:**
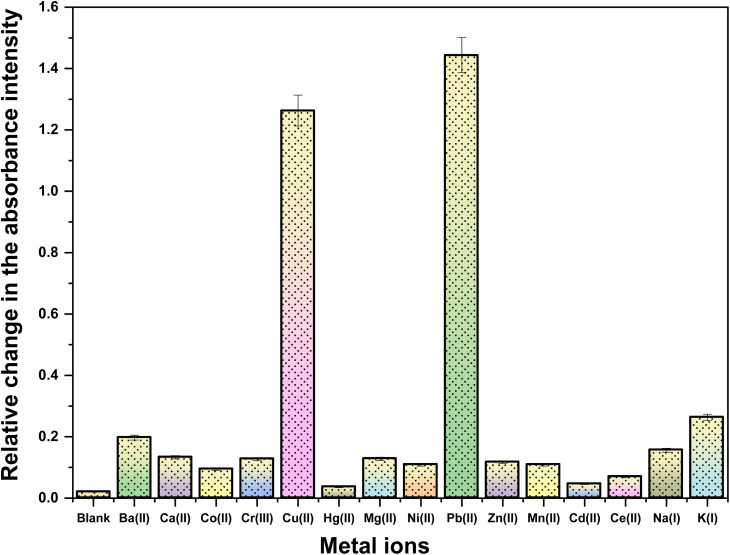
Relative absorption change in the recorded chemosensing behaviour of CBT with different cations.

The ion recognition potential of CBT was scrutinized using UV-visible spectroscopy, wherein DMSO was selected as an ideal solvent confirmed by the ^1^H NMR spectrum of the probe CBT (Fig. S9†), recorded in DMSO and the results were suggestive that the product is stable in this solvent., with 0.05 mM solution of CBT displaying absorption maxima (*λ*_max_) at 348 nm. The chemoselectivity of the probe CBT was tested using the metal chloride solutions (1 mM) of Ba(ii), Ca(ii), Co(ii), Cr(iii), Cu(ii), Hg(ii), Mg(ii), Ni(ii), Pb(ii), and Zn(ii), Mn(ii), Cd(ii), Ce(iii), Na(i), K(i) in DMSO. For the above-listed ions, titration of 0.05 mM CBT solution with 1 mM solutions of the ions exhibit negligible absorption change (Fig. 1), except for Pb(ii) and Cu(ii) ions, which exhibited maximum shifts in absorbance peaks.

#### 3.3.1. Response of CBT towards Pb(ii) and Cu(ii) ions

**Fig. 2:**
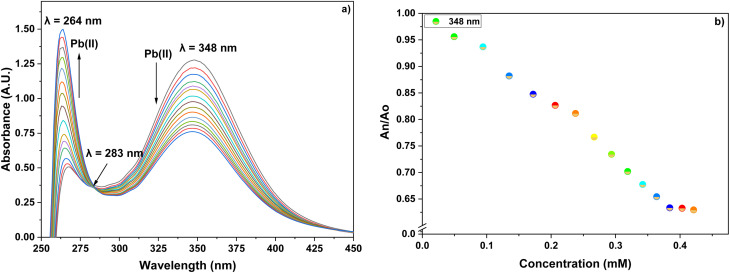
(a) The observed absorption spectrum with the successive addition of 15 equiv. of 1 mM Pb(ii) ions solution in 0.05 mM solution of CBT; (b) the relative change in the absorption intensity on stepwise addition of Pb(ii) ions.

**Fig. 3:**
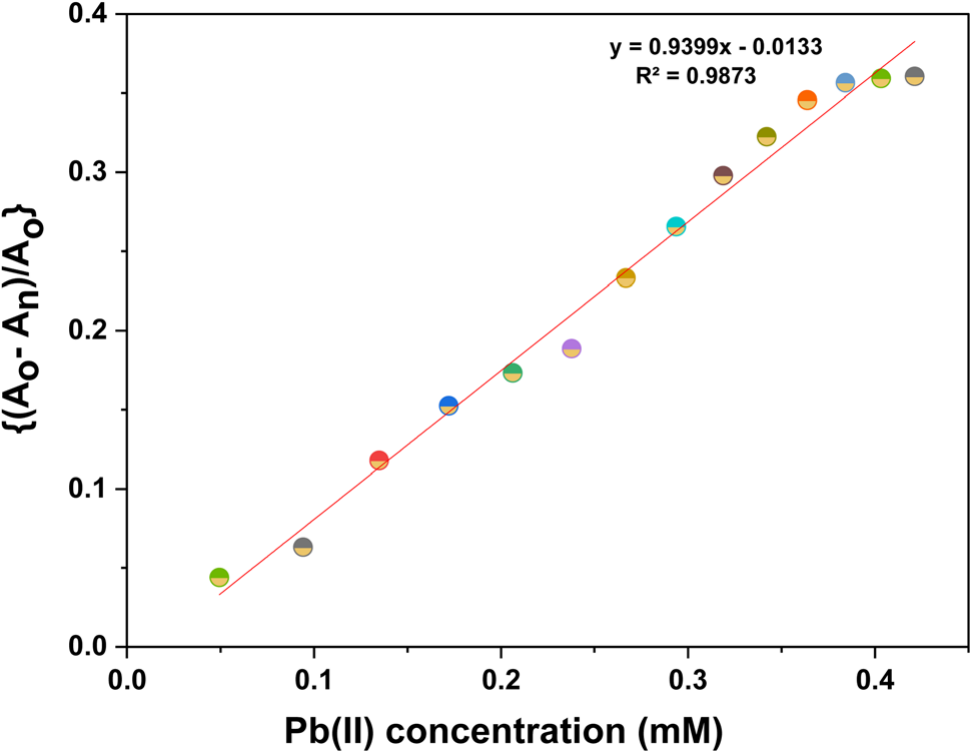
Correlation plot of ((*A*_o_ − *A*_n_)/*A*_o_) *vs.* concentration of Pb(ii) ions, wherein, *A*_o_ = absorbance maxima of CBT complex; *A*_n_ = absorbance maxima on the addition of ions to CBT complex.

**Fig. 4:**
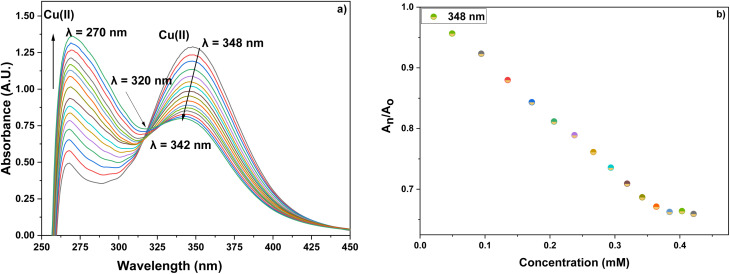
(a) The observed absorption spectrum with the successive addition of 15 equiv. of 1 mM Cu(ii) ion solution in 0.05 mM solution of CBT; (b) the relative change in the intensity on stepwise addition of Cu(ii) ions solution.

**Fig. 5:**
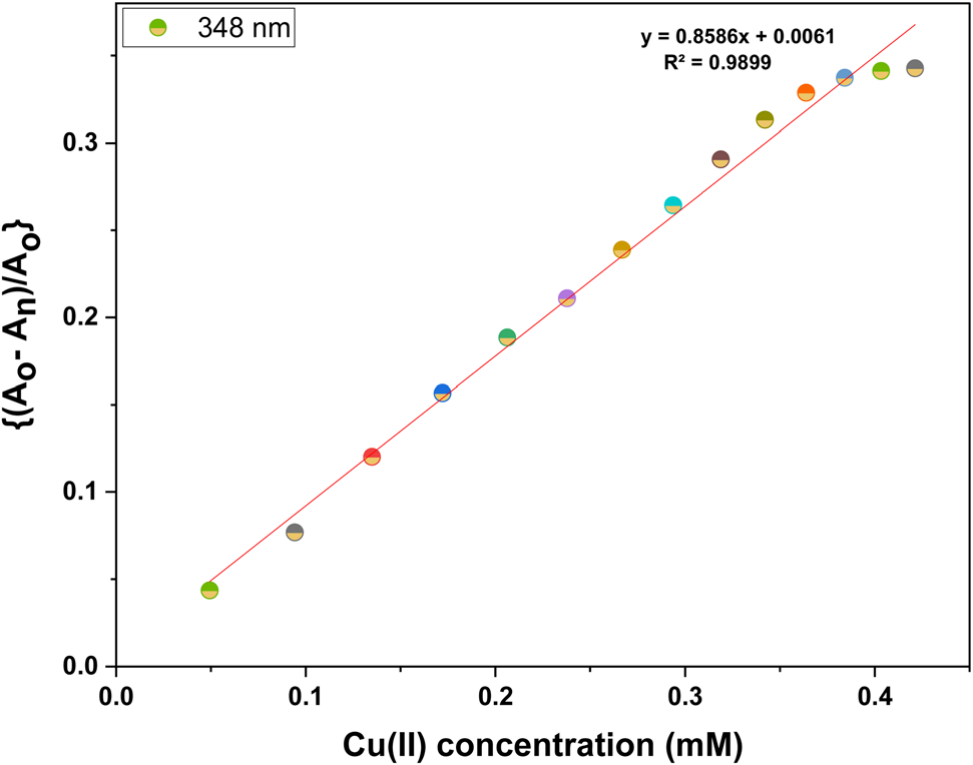
Correlation plot of ((*A*_o_ − *A*_n_)/*A*_o_) *vs.* concentration of Cu(ii).

UV-visible spectroscopic analysis was conducted by titrating CBT with Pb(ii) and Cu(ii) solutions in separate titrations. With the gradual addition of 1 mM Pb(ii) solution, the peak at 348 nm exhibited a hypochromic shift whereas the small shoulder peak at 264 nm witnessed an intense hyperchromic shift, with the production of isosbestic point at 283 nm. The absorption spectrum of CBT upon incremental addition of Pb(ii), and its corresponding relative change in the absorption intensity is shown in Fig. 2(a) and (b), whereas the correlation plot for the same has been shown in Fig. 3. In the case of Cu(ii), the progressive addition of the metal ion solution resulted in a blue shift of about 6 nm in the absorption spectrum from 348 nm to 342 nm with a hypochromic shift. Simultaneously, a significant hyperchromic shift at 270 nm was observed yielding a relatively broad peak with an isosbestic point at 320 nm. The absorption spectrum of CBT with Cu(ii) and its corresponding relative absorption change is shown in Fig. 4(a) and (b), respectively, whereas the correlation plot for the same has been shown in Fig. 5.

**Fig. 6:**
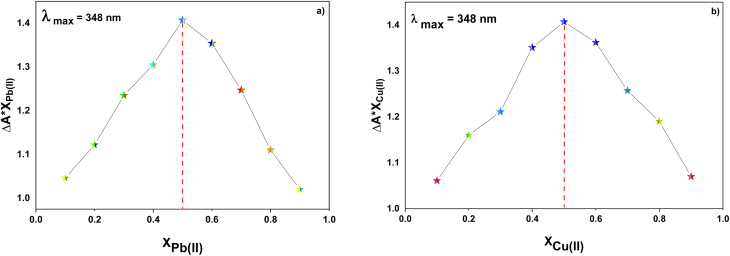
Job's plot analysis of CBT on interaction with (a) Pb(ii) ions and (b) Cu(ii) ions.

Furthermore, the absorption profiles of the individual cations have been included in the ESI (Fig. S14†), and the comparison of the absorption profiles of the sensed metal ions, *i.e.*, Pb(ii) and Cu(ii) with the absorption in Fig. 2(a) and 4(a) further confirms the selective ion binding potential of the chemosensor, owing to the shift in the absorption intensity in accordance with the ligand concentration only, and additionally the appearance of an isosbestic point in the absorption spectrum of CBT on titration with either of the metal ions, thereby indicating the chemical reaction in process and presence of only two species in equilibrium, *i.e.*, the free CBT and metal-bound CBT. Moreover, the analysis of the correlation plots between {(*A*_o_ − *A*_n_)/*A*_o_} and concentration (Fig. 3 and 5) of Pb(ii) and Cu(ii) ions, respectively revealed the detection limit values to be 0.10 mM and 0.11 mM, respectively, and quantification limit values to be 0.35 mM and 0.37 mM, respectively as shown in Table 1. A 1 : 1 metal-to-ligand binding ratio was confirmed for both Pb(ii) and Cu(ii) ions *via* Job's plot as shown in Fig. 6(a) and (b).

**Table 1:** LOD, LOQ, and stoichiometric values of CBT on the addition of Pb(ii) and Cu(ii) ions

Probe	Metal ions	LOD (μM)	LOQ (nM)	Stoichiometry
CBT	Pb(ii)	100	0.35	1 : 1
	Cu(ii)	110	0.37	1 : 1

Furthermore, the information presented in Table 2 provides a comparative analysis of the limit of detection between the chemosensors synthesized previously and the current research, which employs 1,4-disubstituted 1,2,3-triazoles for the detection of Pb(ii) and Cu(ii) ions, as the limit of detection plays a pivotal role in environmental monitoring, especially concerning permissible limits for metal ions set by the Environmental Protection Agency (EPA). It represents the lowest concentration at which a particular metal ion can be reliably detected and quantified, ensuring the accuracy and precision of analytical methods. Meeting or surpassing EPA's specified LODs is crucial in safeguarding public health and the environment by enabling the identification and regulation of potentially harmful metal contaminants in air, water, and soil, thereby facilitating effective pollution control and risk management strategies.^26^

**Table 2:** The compilation of data from chemosensors previously developed for Pb(ii) and Cu(ii)

Entry	Chemosensor	Structure	Metal ion sensed	Limit of detection	Reference
1	Rhodamine B based bis triazole chemosensor	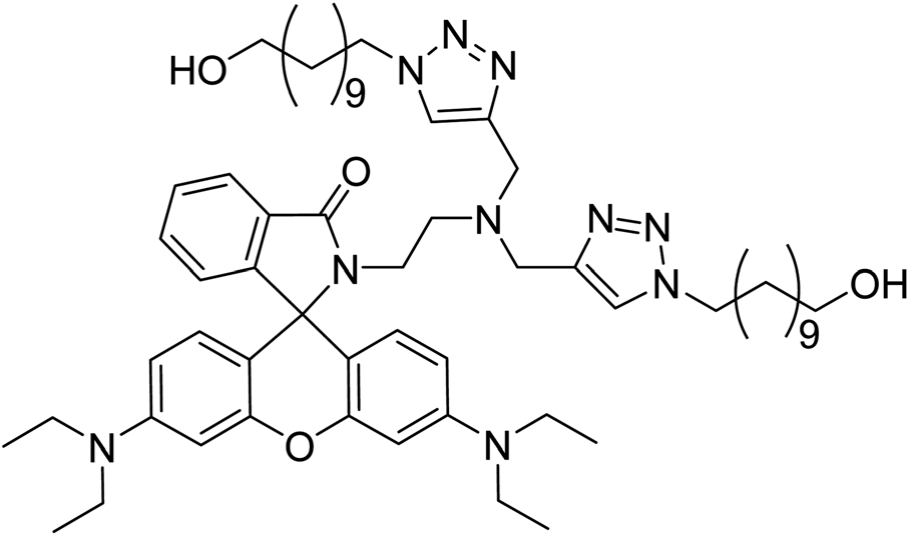	Cu(ii)	100 μM	27
2	Maleic hydrazide based chemosensor	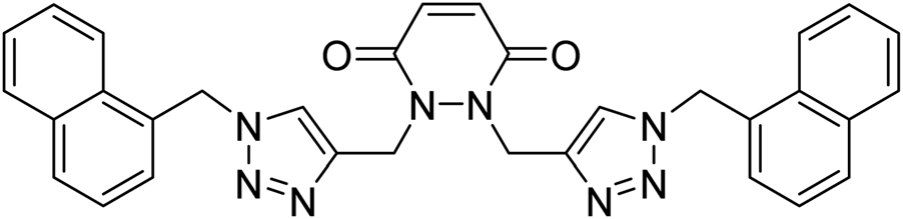	Pb(ii)	142 μM	28
3	APT based chemosensor	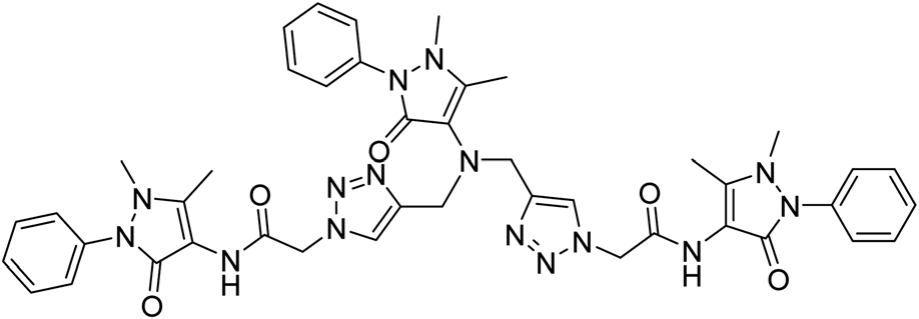	Cu(ii)	63 μM	29
4	CBT chemosensor	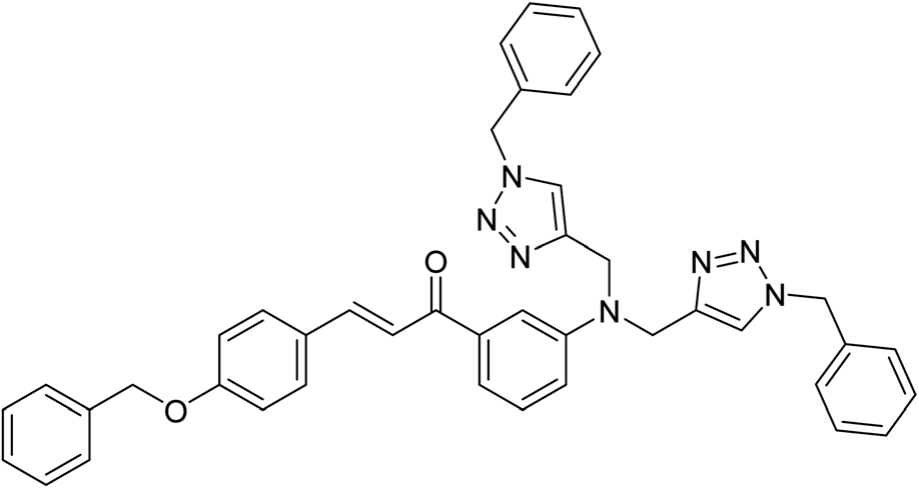	Pb(ii),Cu(ii)	100 μM, 110 μM	This work

#### 3.3.2. Time-dependent analysis of CBT with metal ions

The influence of time on the binding of CBT with Pb(ii) and Cu(ii) ions, were analyzed for 30 minutes and the results obtained indicated the time-independent behavior of CBT on binding with either of the two metal ions. The obtained spectral findings (ESI, Fig. S12 and S13†), represented no change in the binding intensity of the probe over a range of time, as indicated by the straight-lined graph parallel to *x*-axis. It is also worth mentioning that the incremental addition of either of the abovementioned ions to the solution of CBT results in instantaneous and rapid changes in the absorption intensity of the CBT.

#### 3.3.3. Competitive metal ion titration

**Fig. 7:**
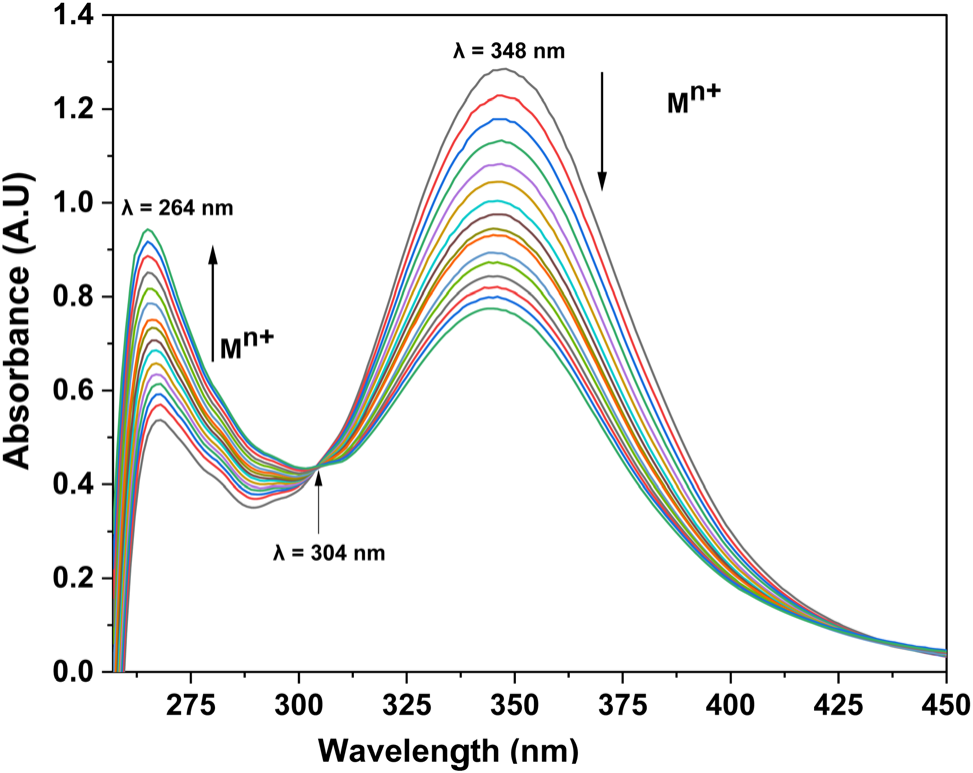
Competitive metal ion titration graph depicting selective recognition of Pb(ii) in the existence of other metal ions.

The competitive ion titration using a 0.05 mM probe solution of CBT in DMSO as the solvent against a solution containing an equimolar concentration of multiple metal ions verified the probe's practical usefulness to preferentially detect Pb(ii) independent of the presence of other metal ions. This was recognized by the observed absorption spectra (Fig. 7) which tend to exhibit similar results as in the spectra of Pb(ii) only. Furthermore, the isosbestic point in case of competitive metal ion titration shifted to 304 nm instead of 283 nm as observed in the case of pure Pb(ii). This shift of 20 nm may be attributed to a combined effect of the change in the molar absorptivity of ligand and a change in the concentration of metal ion in an equimolar solution of all metal ions.

### 3.4. Synthesis and confirmation of CBT-metal complex: ^1^H NMR

After confirming the binding ratio between the ligand CBT and the metal ions *via* Job's plot, the metal–ligand complex corresponding to CBT was synthesized by dissolving the CBT and the metal chloride (1 : 1 by moles) in CHCl_3_/MeOH (1 : 1) and subsequently refluxing the reaction mixture for 4 h. A clear indication of the successful synthesis of the desired product was obtained due to the color change of the solution after refluxing. After filtering the solution, the solvent was evaporated under vacuum. The crude product was scrapped off and characterized *via*^1^H NMR analysis, wherein the formation of the complex through the interaction of the metal atom with the N atoms of both the triazole moieties was evidenced by the spectroscopic analysis as shown in ESI (Fig. S15†). The downfield shifts of the peaks from *δ* = 7.67 ppm corresponding to the 1,2,3-triazole ring proton; and from *δ* = 4.73 ppm and *δ* = 5.54 ppm corresponding to the CH_2_ protons adjacent to either side of both the 1,2,3-triazole moieties in the ^1^H NMR spectrum of CBT to *δ* = 7.79 ppm, *δ* = 5.02 ppm and *δ* = 5.62 ppm, respectively, in the ^1^H NMR spectrum of the CBT–metal complex supported the binding of the metal atom with the 1,2,3-triazole moiety. Furthermore, the DFT analysis of the metal–ligand complex was also undertaken by applying the 631G+(d,p) basis set for CBT; whereas the LANL2DZ basis set was applied for the metal atom which also reinforced the experimental results by exhibiting binding interactions between the metal atom and the N atoms of both the triazole moieties.

## 4. Computational details

### 4.1. Ligand optimization (DFT)

**Fig. 8:**
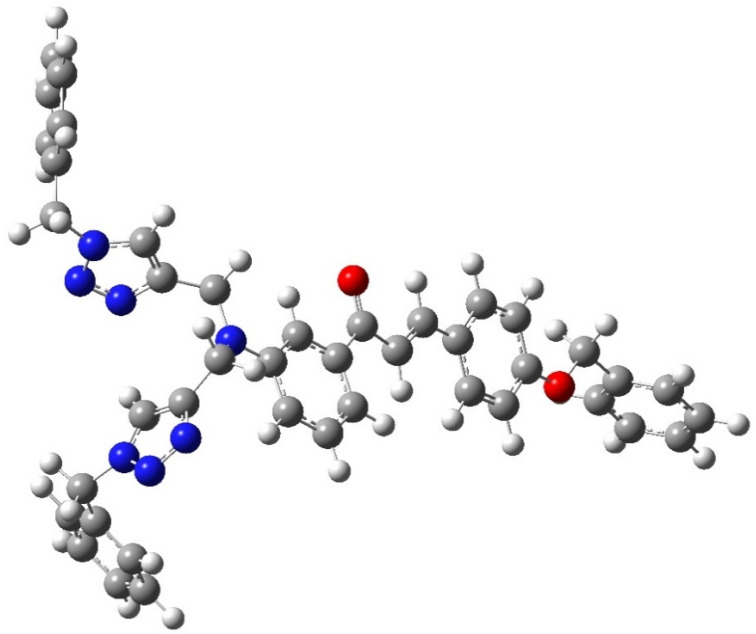
The optimized structure of CBT probe using DFT (B3LYP/631G+(d,p) basis set).

**Fig. 9:**
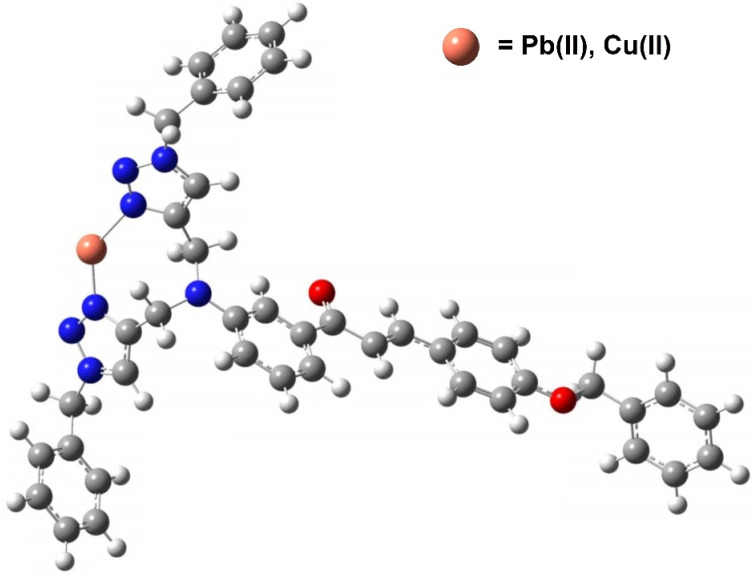
The binding mode of CBT, through the 1,2,3-triazole moiety, with the metal ions as studied by B3LYP/LANL2DZ basis set using Gaussian 09 software.

**Fig. 10:**
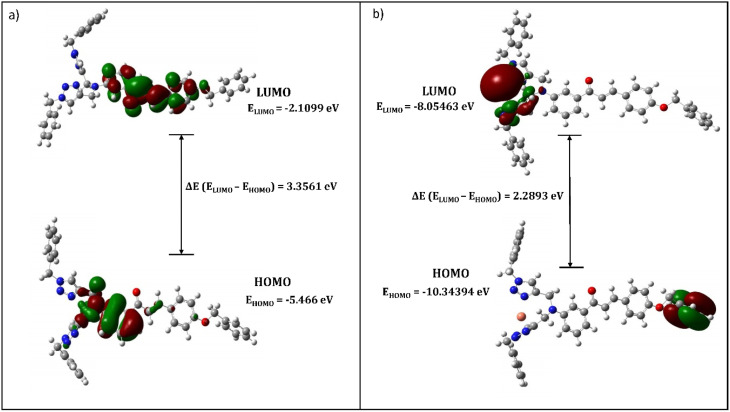
Pictorial representation of HOMO–LUMO (a) CBT (b) CBT-metal complex.

The structural insights about complex structures with extensive conjugation can be gleaned from quantum chemical simulations using density functional theory (DFT). The simulations based on DFT were used to perceive the special aspects of the energy-optimized structure of the synthesized probe CBT employing the B3LYP/631G+(d,p) basis set for C, H, O, N and the B3LYP/LANL2DZ set for the metal. The energy-minimized structure of CBT and the CBT–metal complex was obtained and analyzed using Gaussian 09 software package.^30^Fig. 8 depicts the optimized structure of CBT (*Cartesian coordinates are given in the ESI, Table S1*†), and Fig. 9 depicts the structural aspects of CBT–metal complexation, wherein the simulations are suggestive of interaction between the probe and the metal ion *via* the lone pairs of the N atoms of the 1,2,3-triazole rings (one N atom from each 1,2,3-triazole moiety). In addition, Fig. 10(a) and (b) depicts the energy difference between the HOMO and the LUMO densities over the molecular structure for both CBT and CBT–metal complex respectively. HOMO (π donor) in case of CBT is delocalized adjoining the 1,2,3-triazole rings, while LUMO (π acceptor) is delocalized on the electron withdrawing carbonyl group, whereas the metal ion and the 1,2,3-triazole ring have a delocalized LUMO electron density. Compared to the free CBT, the CBT-metal complex has a lower Δ*E* value of 2.2893 eV, indicating improved stability of the complex (Table 3).

**Table 3:** Calculated electronic data of CBT and metal complex

Parameters	CBT	CBT–metal complex
*E*_HOMO_ (eV)	−5.466	−10.3439
*E*_LUMO_ (eV)	−2.1099	−8.05463
*E*_g_ (eV)	3.3561	2.2893
Charge	0	2
Dipole moment (Debye)	4.1998	10.1056
Point group	C1	C1

### 4.3. Molecular electrostatic potential (MEP) analysis

**Fig. 11:**
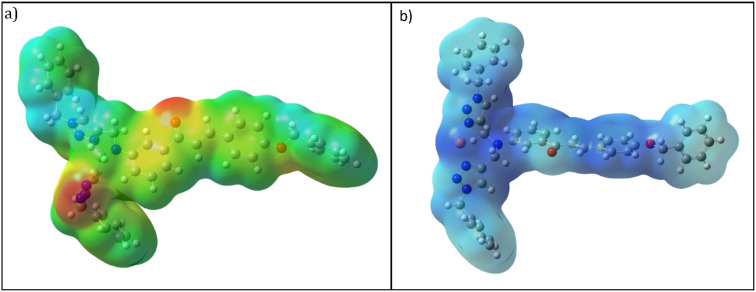
Molecular electrostatic potential (MEP) map (a) CBT (b) CBT–metal complex.

It is crucial to identify electrophilic and nucleophilic sites in the molecule before making any predictions about the molecule's reactivity.^31,32^ The MEP is used to identify the preferred positions for electrophilic and/or nucleophilic attack. The MEP scale has a color bar from red > orange > yellow > green > blue indicating the negative to positive potential values. Red has the most negative electrostatic potential value and *vice versa* for blue color. In the case of CBT, as shown in Fig. 11(a), the electron-rich triazole rings and carbonyl group simultaneously appeared red having a negative electrostatic potential and availability for intermolecular interactions. Further, Fig. 11(b) demonstrates that the N atom of the 1,2,3-triazole ring binds with the metal ion, thereby reducing its electron density.

## 5. Molecular docking

**Fig. 12:**
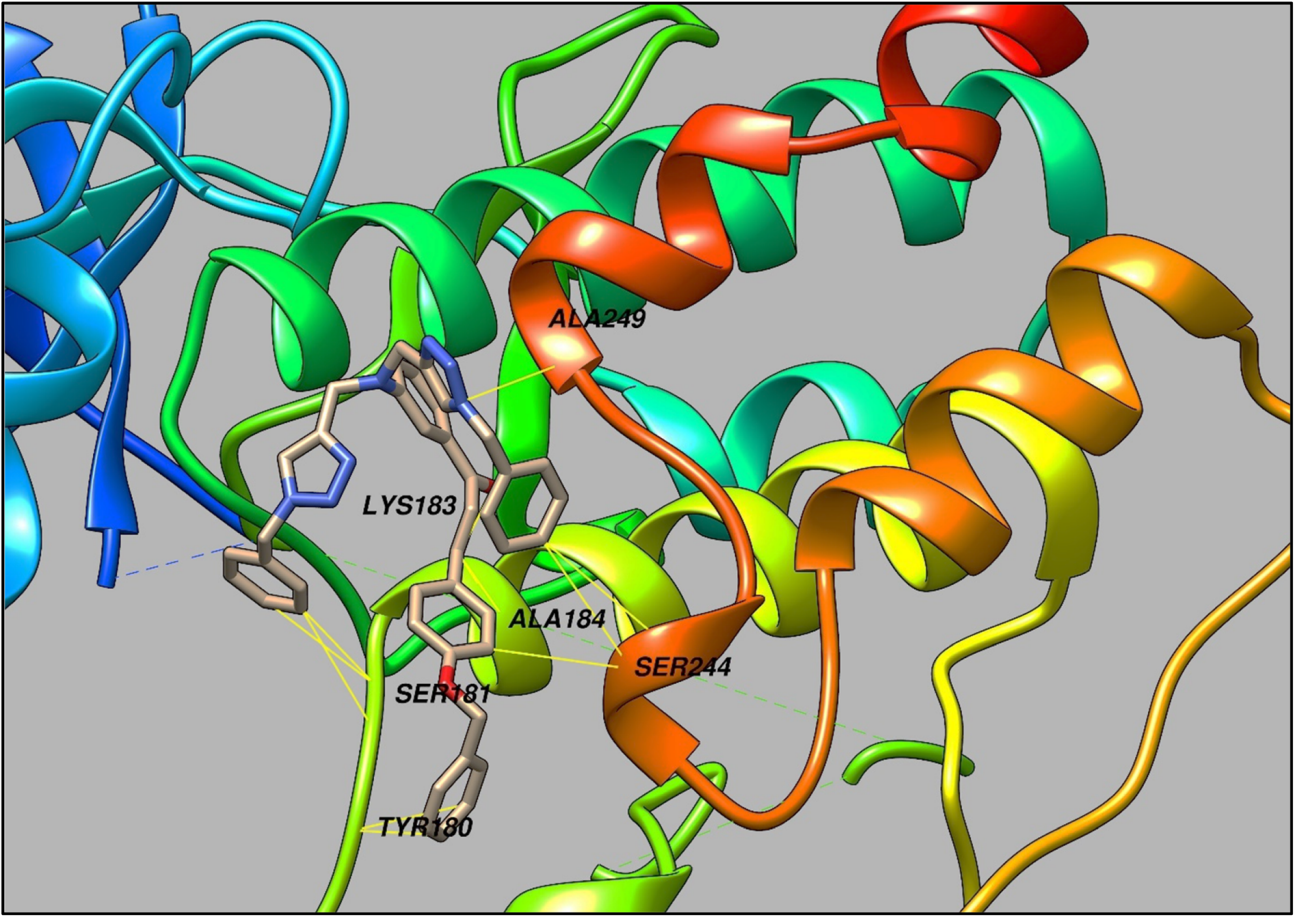
A representation of the binding mode of CBT with TRIB1 protein through several amino acid residues (*Envisioned through UCSF chimera software*).^39^

The 1,2,3- triazole moiety-containing compounds are rapidly becoming a focal point in the design and synthesis of bioactive compounds, which have been linked to an inclusive range of biological activities such as antibacterial, antifungal, anti-inflammatory, anticonvulsant, anti-HIV, antineoplastic, and antiproliferative effects.^33–35^ Keeping in view this aspect, the synthesized CBT probe was predicted for its pharmacological properties using the Way2drug platform, a highly cited and trusted prediction tool for analyzing the potential of organic probes in pharmacology.^36^ The results so obtained were suggestive of the atherosclerosis inhibitory effect of the probe, and hence in order to investigate the potential of CBT as atherosclerosis inhibitor, it was subsequently docked to the TRIB1 protein using Auto Dock Vina.^37,38^ As represented in Fig. 11, CBT exhibited interactions with the protein *via* multiple amino acid residues such as TYR180, SER181, LYS183, ALA184, SER244, ALA249, *etc.*, and also demonstrated a high binding affinity with the value of −6.2 kcal mol^−1^ (Fig. 12).

## 6. Plausible mode of interaction

**Fig. 13:**
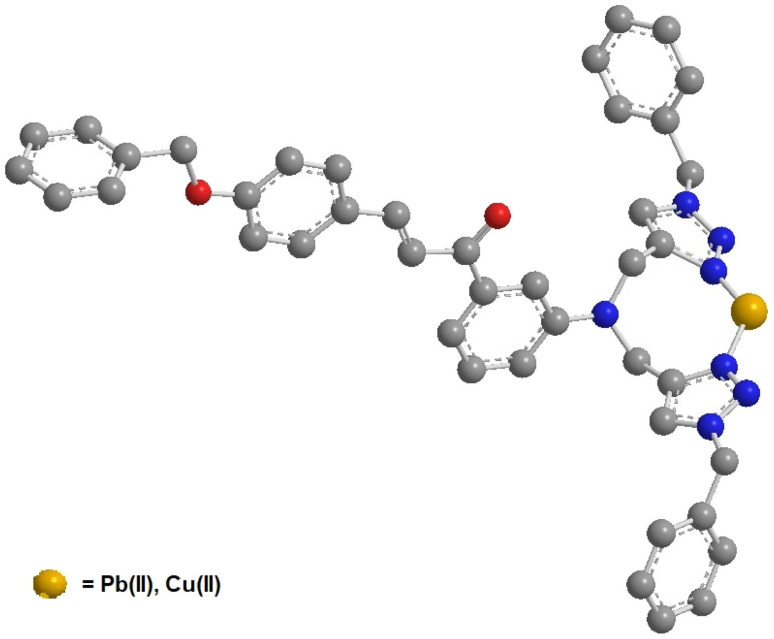
Plausible binding mode of CBT with metal ion *via* nitrogen atoms of both triazole rings (*H atoms have been omitted for clarity*).

Using the hard soft acid base (HSAB) framework, Pb(ii) and Cu(ii) are categorized as weak acids. Both metal ions may interact with the lone-pair-carrying atomic groups like N, O, or S.^40^ Therefore, in accordance to the HSAB concept, the CBT probe can capture the incoming electron deficient metal ions by interacting *via* the lone pair-bearing N atoms of both the 1,2,3-triazole moieties. The same prediction is experimentally validated by the DFT calculations of the CBT-metal complex are also suggestive of CBT-metal binding *via* the N atoms of the 1,2,3-triazole rings. On the basis of these observations, a plausible binding mode that demonstrates a 1 : 1 stoichiometry of ligand–metal is illustrated in Fig. 13.

## 7. Conclusion

The 'CuAAC' based synthesis of chalcomer containing 1,2,3-triazole based chemosensor (CBT) and was characterized using standard spectroscopic techniques such as IR, NMR (^1^H and ^13^C), and mass spectrometry. The probe was determined to have selective recognition behavior for Pb(ii) and Cu(ii) ions, two of the several metal ions known to cause insidious toxicity to humans on accumulation in significant concentrations in the body. In addition to this, the competitive metal ion titrations revealed the preferential binding of CBT for Pb(ii) ions, even in the presence of multiple ions. The 1 : 1 metal-to-ligand stoichiometric ratio was observed for Pb(ii) and Cu(ii) ions, as shown in Job's plot. The time-independent behavior of CBT was evidenced by its binding to Pb(ii) and Cu(ii) ions resulting in a straight line with no change in binding strength over time, thereby confirming its robust binding potential. In addition, the unique molecular arrangement of the probe was identified by DFT analyses, and the molecule's electronic properties corresponding to the spatial orientation are reported. Furthermore, CBT was explored for its anti-atherosclerosis capability by performing docking analysis on the TRIB1 protein, which is known to cause early atherosclerosis.

## Author contributions

Riddima Singh: writing – original draft, investigation, formal analysis. Gurleen Singh: data curation, methodology, resources. Nancy George: data curation, methodology. Gurjaspreet Singh: visualization, validation. Anita Devi: formal analysis, resources. Harminder Singh: conceptualization, validation. Gurpreet Kaur: investigation, writing – review & editing, supervision. Jandeep Singh: conceptualization, supervision.

## Conflicts of interest

There are no conflicts to declare.

## Supplementary Material

RA-013-D3RA05760G-s001

## References

[cit1] Briffa J., Sinagra E., Blundell R. (2020). Heavy metal pollution in the environment and their toxicological effects on humans. Heliyon.

[cit2] Wu Y., Pang H., Liu Y., Wang X., Yu S., Fu D., Chen J., Wang X. (2019). Environmental remediation of heavy metal ions by novel-nanomaterials: a review. Environ. Pollut..

[cit3] Bansod B., Kumar T., Thakur R., Rana S., Singh I. (2017). A review on various electrochemical techniques for heavy metal ions detection with different sensing platforms. Biosens. Bioelectron..

[cit4] Zheng Q., Li Q., Hu S., Guo X., Yang H. (2023). A highly selective SERS chip for rapid detection of copper ions in aquatic system. J. Environ. Chem. Eng..

[cit5] Singh G., George N., Singh R., Singh G., Kaur J. D., Kaur G., Singh H., Singh J. (2022). CuAAC-Derived Selective Fluorescent Probe as a Recognition Agent for Pb(II) and Hg(II): DFT and Docking Studies. ACS Omega.

[cit6] Collin M. S., Venkatraman S. K., Vijayakumar N., Kanimozhi V., Arbaaz S. M., Stacey R. G. S., Anusha J., Choudhary R., Lvov V., Tovar G. I., Senatov F., Koppala S., Swamiappan S. (2022). Bioaccumulation of lead (Pb) and its effects on human: a review. J. Hazard. Mater. Adv..

[cit7] Agrahari A. K., Kumar S., Pandey M. D., Rajkhowa S., Jaiswal M. K., Tiwari V. K. (2022). Click Chemistry - Inspired Synthesis of Porphyrin Hybrid Glycodendrimers as Fluorescent Sensor for Cu(II) Ions. ChemistrySelect.

[cit8] Tarnowska M., Krawczyk T. (2020). Click chemistry as a tool in biosensing systems for sensitive copper detection. Biosens. Bioelectron..

[cit9] Cao F., Jiao F., Ma S., Dong D. (2022). Laser-induced breakdown spectroscopy mediated amplification sensor for copper (II) ions detection using click chemistry. Sens. Actuators, B.

[cit10] Ge C., Li J., Wang D., Lv K., Liu Q., Shen Y., Zhuang X., Luo W., Wu Z., Zhang Y., Shi L., Liu L., Bao S., Zhang H. (2021). Graphdiyne nanosheets as a platform for accurate copper(ii) ion detection via click chemistry and fluorescence resonance energy transfer. RSC Adv..

[cit11] Pandey N., Jyoti, Singh M., Dwivedi P., Sahoo S. C., Mishra B. B. (2022). Click chemistry inspired synthesis of andrographolide triazolyl conjugates for effective fluorescent sensing of ferric ions. Nat. Prod. Res..

[cit12] Abdullah A., Nuri Kursunlu A., Guler E. (2023). A high-performance fluorescent hybrid material for fluorometric detection and removal of toxic Pb(ii) ions from aqueous media: performance and challenges. RSC Adv..

[cit13] Singh G., Sushma, Singh A., Satija P., Shilpy, Mohit, Priyanka, Singh J., Khosla A. (2021). Schiff base derived bis-organosilanes: immobilization on silica nanosphere and Cu^2+^ and Fe^3+^ dual ion sensing. Inorganica Chim. Acta.

[cit14] Ahmed F., Xiong H. (2021). Recent developments in 1,2,3-triazole-based chemosensors. Dyes Pigments.

[cit15] Elkanzi N. A. A., Hrichi H., Alolayan R. A., Derafa W., Zahou F. M., Bakr R. B. (2022). Synthesis of Chalcones Derivatives and Their Biological Activities: A Review. ACS Omega.

[cit16] Jasim H. A., Nahar L., Jasim M. A., Moore S. A., Ritchie K. J., Sarker S. D. (2021). Chalcones: Synthetic Chemistry Follows Where Nature Leads. Biomolecules.

[cit17] Suyambulingam A., Nair S., Chellapandian K. (2022). Synthesis, spectral characterization of novel chalcones based oxazines derivatives and screening of their antimicrobial and antioxidant activity. J. Mol. Struct..

[cit18] Singh R., Singh G., George N., Singh G., Gupta S., Singh H., Kaur G., Singh J. (2023). Copper-Based Metal–Organic Frameworks (MOFs) as an Emerging Catalytic Framework for Click Chemistry. Catalysts.

[cit19] Singh G., Majeed A., Singh R., George N., Singh G., Gupta S., Singh H., Kaur G., Singh J. (2023). CuAAC ensembled 1,2,3-triazole linked nanogels for targeted drug delivery: a review. RSC Adv..

[cit20] Singh G., Singh J., Mangat S. S., Arora A. (2014). Synthetic approach towards ‘click’ modified chalcone based organotriethoxysilanes; UV-Vis study. RSC Adv..

[cit21] Meldal M., Diness F. (2020). Recent Fascinating Aspects of the CuAAC Click Reaction. Trends Chem..

[cit22] Meldal M., Tornøe C. W. (2008). Cu-Catalyzed Azide−Alkyne Cycloaddition. Chem. Rev..

[cit23] Barone , Petersson G. A., Nakatsuji H., Li X., Caricato M., Marenich A., Bloino J., Janesko B. G., Gomperts R., Mennucci B., Hratchian H. P., Ortiz J. V., Izmaylov A. F., Sonnenberg J. L., Williams-Young D., Ding F., Lipparini F., Egidi F., Goings J., Peng B., Petrone A., Henderson T., Ranasinghe D., Zakrzewski V. G., Gao J., Rega N., Zheng G., Liang W., Hada M., Ehara M., Toyota K., Fukuda R., Hasegawa J., Ishida M., Nakajima T., Honda Y., Kitao O., Nakai H., Vreven T., Throssell K., Montgomery Jr J. A., Peralta J. E., Ogliaro F., Bearpark M., Heyd J. J., Brothers E., Kudin K. N., Staroverov V. N., Keith T., Kobayashi R., Normand J., Raghavachari K., Rendell A., Burant J. C., Iyengar S. S., Tomasi J., Cossi M., Millam J. M., Klene M., Adamo C., Cammi R., Ochterski J. W., Martin R. L., Morokuma K., Farkas O., Foresman J. B. and Fox D. J., GAUSSIAN 09 (Revision A.02), Gaussian Inc., Wallingford CT, 2016

[cit24] Karbasi M. M., Mirjafary Z., Saeidian H., Mokhtari J. (2021). Efficient synthesis and DFT analysis of novel 1,2,3-triazole-based dithiocarbamates. J. Mol. Struct..

[cit25] Stefanello F. S., Kappenberg Y. G., Ketzer A., Franceschini S. Z., Salbego P. R. S., Acunha T. V., Nogara P. A., Rocha J. B. T., Martins M. A. P., Zanatta N., Iglesias B. A., Bonacorso H. G. (2021). New 1-(Spiro[chroman-2,1′-cycloalkan]-4-yl)-1H-1,2,3-triazoles:
synthesis, QTAIM/MEP analyses, and DNA/HSA-binding assays. J. Mol. Liq..

[cit26] U.S. Environmental Protection Agency|US EPA , https://www.epa.gov/, accessed 2023-10-08

[cit27] Rathinam B., Chien C.-C., Chen B.-C., Liu J.-H. (2013). Fluorogenic and Chromogenic Detection of Cu^2+^ and Fe^3+^ Species in Aqueous Media by Rhodamine–Triazole Conjugate. Tetrahedron.

[cit28] Singh G., Singh R., George N., Singh G., Satija P., Kaur G., Singh H., Singh J. (2023). Selective Recognition of Pb(II) and Cr(III) by Novel Maleic Hydrazide-Based 1,2,3-Triazole Linked Derivatives. J. Mol. Struct..

[cit29] Kumar S., Lal B., Tittal R. K., Singh G., Singh J., D G. V., Sharma R., Sabane J. K. (2023). A Selective Chemosensor via Click Chemistry for Cu^2+^ and Hg^2+^ Ions in Organic Media. Sens. Diagn..

[cit30] Frisch M. J. , Trucks G. W., Schlegel H. B., Scuseria G. E., Robb M. A., Cheeseman J. R., Scalmani G., Barone V., Mennucci B., Petersson G. A., Nakatsuji H., Caricato M., Li X., Hratchian H. P., Izmaylov A. F., Bloino J., Zheng G., Sonnenberg J. L., Hada M., Ehara M., Toyota K., Fukuda R., Hasegawa J., Ishida M., Nakajima T., Honda Y., Kitao O., Nakai H., Vreven T., Montgomery Jr J. A., Peralta J. E., Ogliaro F., Bearpark M., Heyd J. J., Brothers E., Kudin K. N., Staroverov V. N., Kobayashi R., Normand J., Raghavachari K., Rendell A., Burant J. C., Iyengar S. S., Tomasi J., Cossi M., Rega N., Millam J. M., Klene M., Knox J. E., Cross J. B., Bakken V., Adamo C., Jaramillo J., Gomperts R., Stratmann R. E., Yazyev O., Austin A. J., Cammi R., Pomelli C., Ochterski J. W., Martin R. L., Morokuma K., Zakrzewski V. G., Voth G. A., Salvador P., Dannenberg J. J., Dapprich S., Daniels A. D., Farkas O., Foresman J. B., Ortiz J. V., Cioslowski J. and Fox D. J., Gaussian 09, Revision B.01, Gaussian Inc., Wallingford, 2010

[cit31] Karbasi M. M., Mirjafary Z., Saeidian H., Mokhtari J. (2021). Efficient Synthesis and DFT Analysis of Novel 1,2,3-Triazole-Based Dithiocarbamates. J. Mol. Struct..

[cit32] Stefanello F. S., Kappenberg Y. G., Ketzer A., Franceschini S. Z., Salbego P. R. S., Acunha T. V., Nogara P. A., Rocha J. B. T., Martins M. A. P., Zanatta N., Iglesias B. A., Bonacorso H. G. (2021). New 1-(Spiro[Chroman-2,1′-Cycloalkan]-4-Yl)-1H-1,2,3-Triazoles: Synthesis, QTAIM/MEP Analyses, and DNA/HSA-Binding Assays. J. Mol. Liq..

[cit33] Marzi M., Farjam M., Kazeminejad Z., Shiroudi A., Kouhpayeh A., Zarenezhad E. (2022). A Recent Overview of 1,2,3-Triazole-Containing Hybrids as Novel Antifungal Agents: Focusing on Synthesis, Mechanism of Action, and Structure-Activity Relationship (SAR). J. Chem..

[cit34] Kaur G., Kumar P. (2022). Ibuprofen Tagged Imine RT-COF-1 as Customisable Vehicle for Controlled Drug Delivery Application. Inorg. Chem. Commun..

[cit35] Kaur G., Kumar D., Sundarrajan S., Ramakrishna S., Kumar P. (2023). Recent Trends in the Design, Synthesis and Biomedical Applications of Covalent Organic Frameworks. Polymers.

[cit36] Druzhilovskiy D. S., Rudik A. V., Filimonov D. A., Gloriozova T. A., Lagunin A. A., Dmitriev A. V., Pogodin P. V., Dubovskaya V. I., Ivanov S. M., Tarasova O. A., Bezhentsev V. M., Murtazalieva K. A., Semin M. I., Maiorov I. S., Gaur A. S., Sastry G. N., Poroikov V. V. (2017). Computational Platform Way2Drug: From the Prediction of Biological Activity to Drug Repurposing. Russ. Chem. Bull..

[cit37] Trott O., Olson A. J. (2010). AutoDock Vina: improving the speed and accuracy of docking with a new scoring function, efficient optimization, and multithreading. J. Comput. Chem..

[cit38] Johnston J. M. , Angyal A., Bauer R. C., Hamby S., Suvarna S. K., Baidžajevas K., Hegedus Z., Dear T. N. and Turner M., The Cardiogenics Consortium10.1126/sciadv.aax9183 PMC6821468 31692955

[cit39] Pettersen E. F., Goddard T. D., Huang C. C., Couch G. S., Greenblatt D. M., Meng E. C., Ferrin T. E. (2004). UCSF Chimera - A Visualization System for Exploratory Research and Analysis. J. Comput. Chem..

[cit40] Pearson R. G. (1963). Hard and Soft Acids and Bases. J. Am. Chem. Soc..

