# Supplementary material for: Cu(i)-catalysed 1,2,3-triazole stitched chalcomer assembly as Pb(ii) and Cu(ii) ion sensor: DFT and docking scrutiny
Source: RSC Adv. 2023 Nov 3;13(46):32399–412. doi: 10.1039/d3ra05760g (PMC10623384; doi:10.1039/d3ra05760g)
Supplement: RA-013-D3RA05760G-s001 [file RA-013-D3RA05760G-s001.pdf]

Electronic Supplementary Material (ESI) for RSC Advances.
This journal is © The Royal Society of Chemistry 2023

Supporting Information

Cu(I)-catalysed 1,2,3-triazole stitched chalcomer assembly as Pb(II) and Cu(II) ion sensor: DFT and docking scrutiny

Riddima Singh,[a] Gurleen Singh,[a] Nancy George,[a] Gurjaspreet Singh,[b] Anita Devi,[b] Harminder Singh,[a] Gurpreet Kaur,[c,#] Jandeep Singh,[a,*]

[a] School of Chemical Engineering and Physical Sciences, Lovely Professional University, Phagwara–144411 (Punjab)

[b] Department of Chemistry and Centre of Advanced Studies in Chemistry, Panjab University, Chandigarh–160014

[c] Department of Chemistry, GGN Khalsa College, Civil lines, Ludhiana–141001 (Punjab)

***,# Corresponding Authors**

Contact details:

***singhjandeep@gmail.com**

Contents:

Figure S1: IR spectrum of chalcone 3

Figure S2: IR spectrum of alkyne 4

Figure S3: ^{1}H NMR spectrum of alkyne 4

Figure S4: ^{13}C NMR spectrum of alkyne 4

Figure S5: IR spectrum of benzyl azide 5

Figure S6: ^{1}H NMR spectrum of benzyl azide 5

Figure S7: ^{13}C NMR spectrum of benzyl azide 5

Figure S8: IR spectrum of probe CBT

Figure S9: ^{1}H NMR spectrum of CBT

Figure S10: ^{13}C NMR spectrum of CBT

Figure S11: Mass spectrum of CBT

Figure S12: Time dependent spectra of CBT-Pb(II) complex displaying the trend in the absorption.

Figure S13: Time dependent spectra of CBT-Cu(II) complex displaying the trend in the absorption.

Figure S14: Individual absorption profile of all cations.

Figure S15: ^{1}H-NMR of probe CBT on interaction with the metal ion.

Table S1: Cartesian co-ordinates of CBT

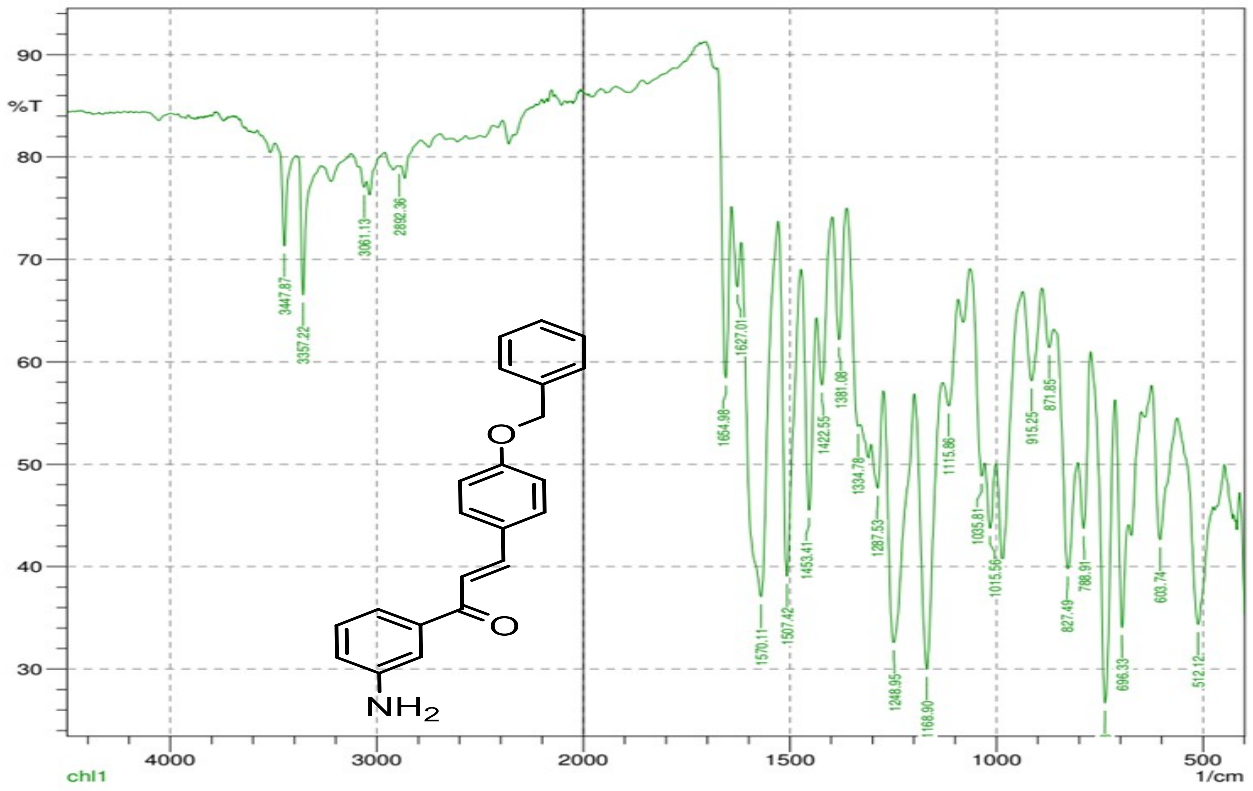

Figure S1: IR spectrum of chalcone 3

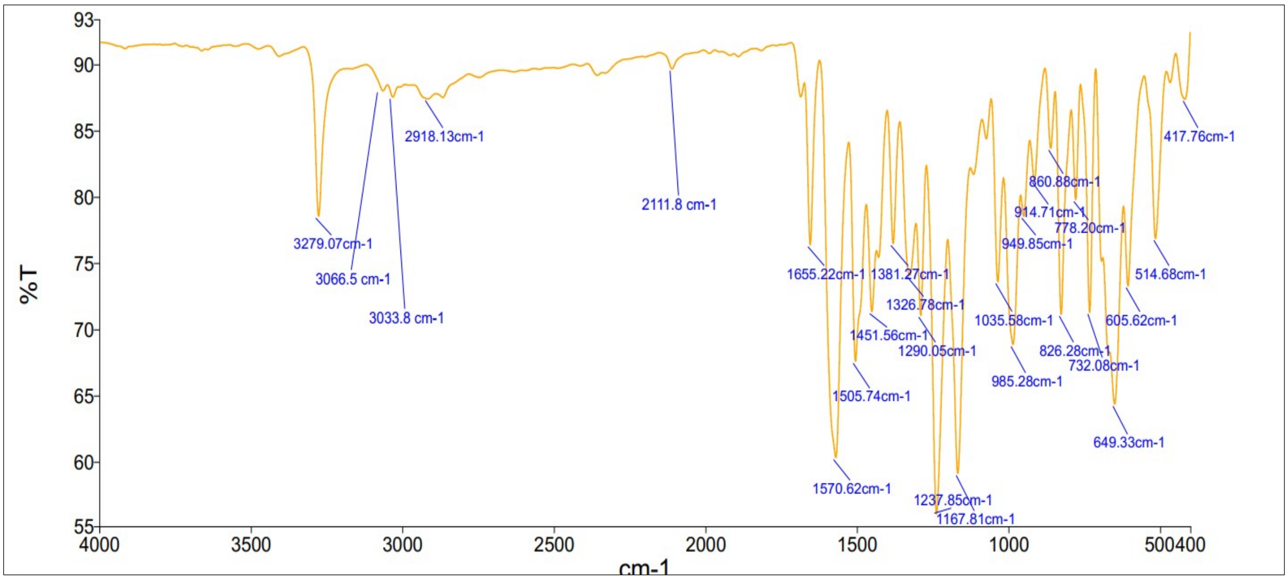

Figure S2: IR spectrum of alkyne 4

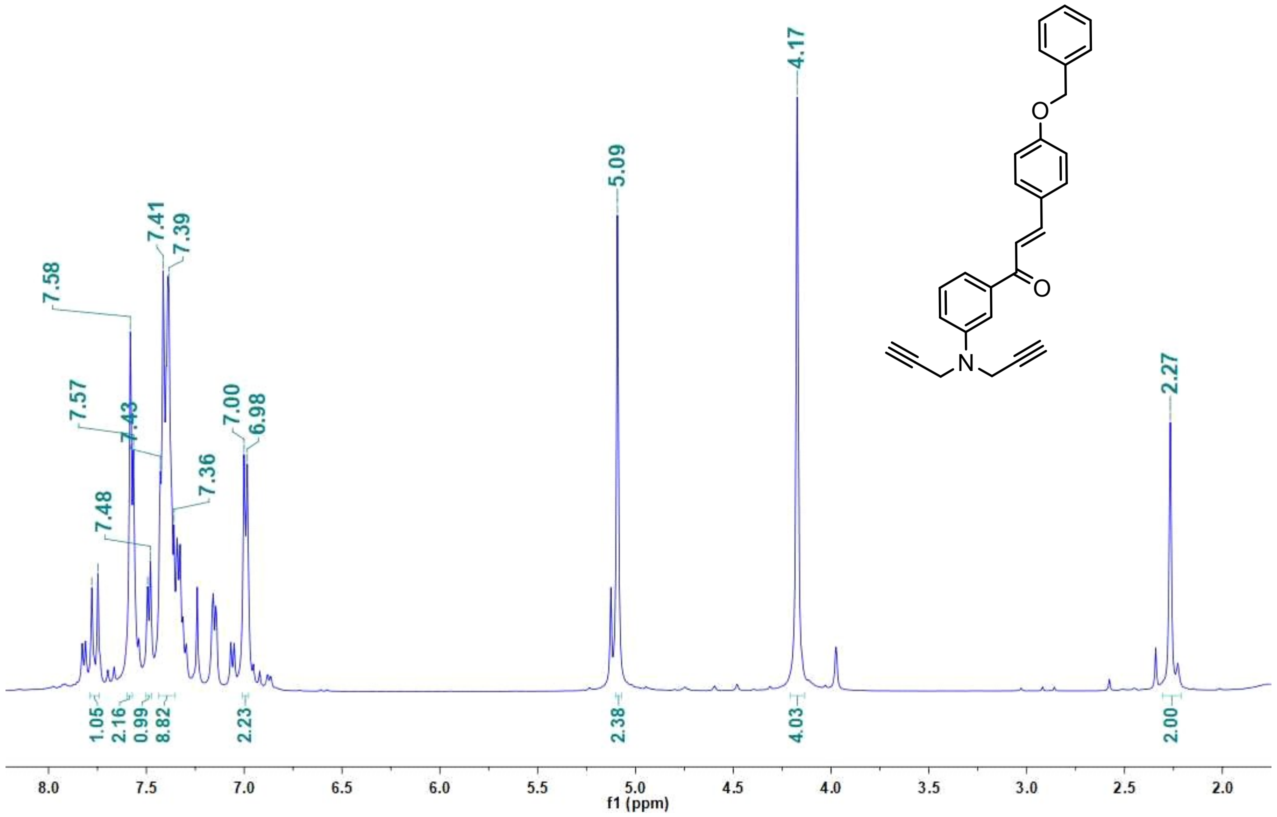

Figure S3: ^{1}H NMR spectrum of alkyne 4

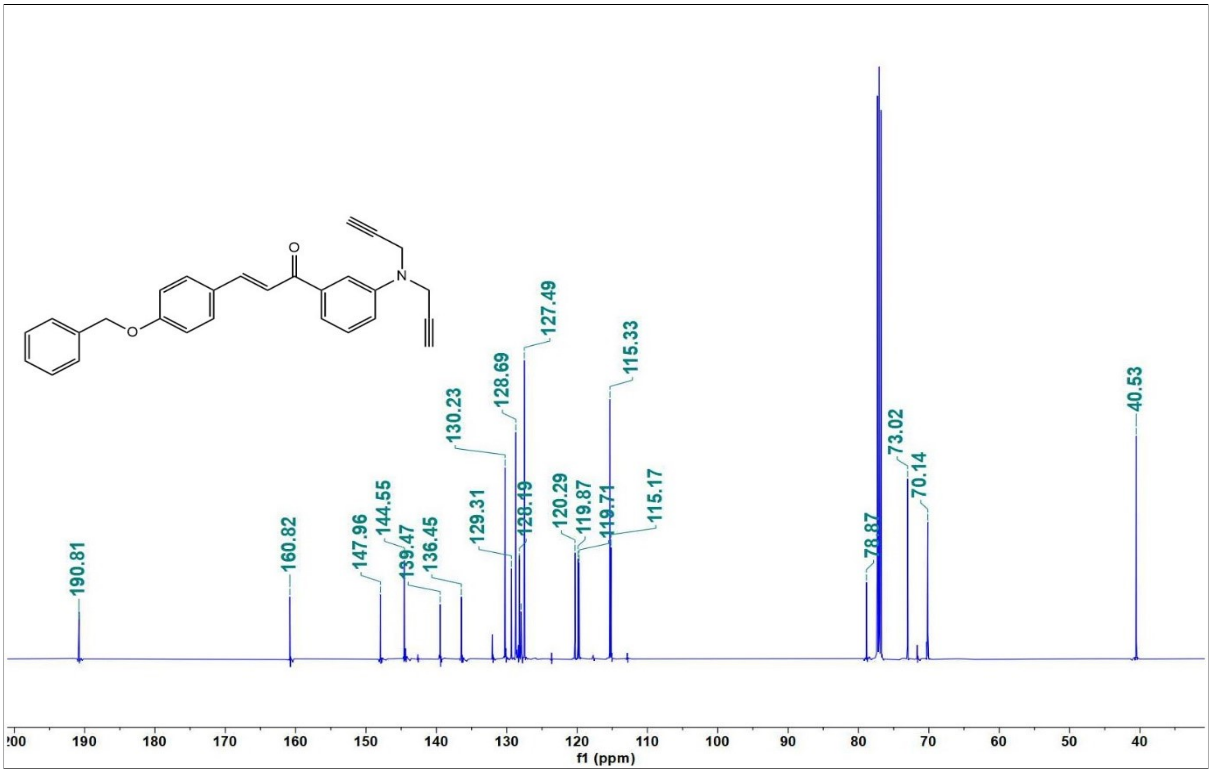

Figure S4: ^{13}C NMR spectrum of alkyne 4

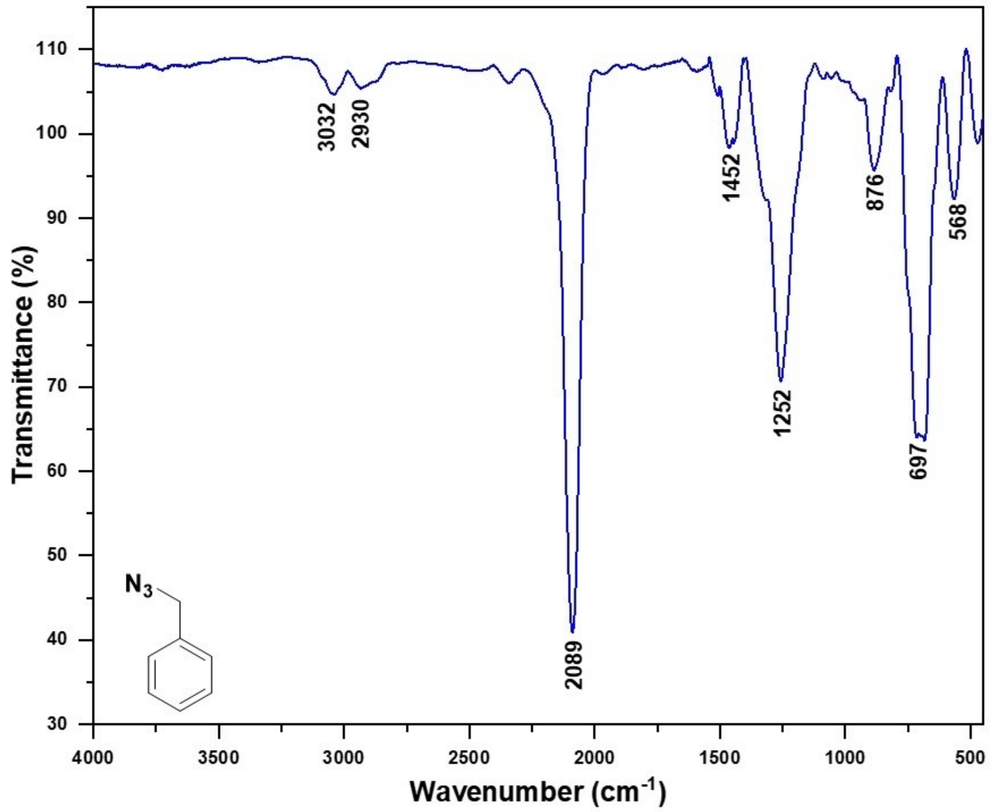

Figure S5: IR spectrum of benzyl azide 5

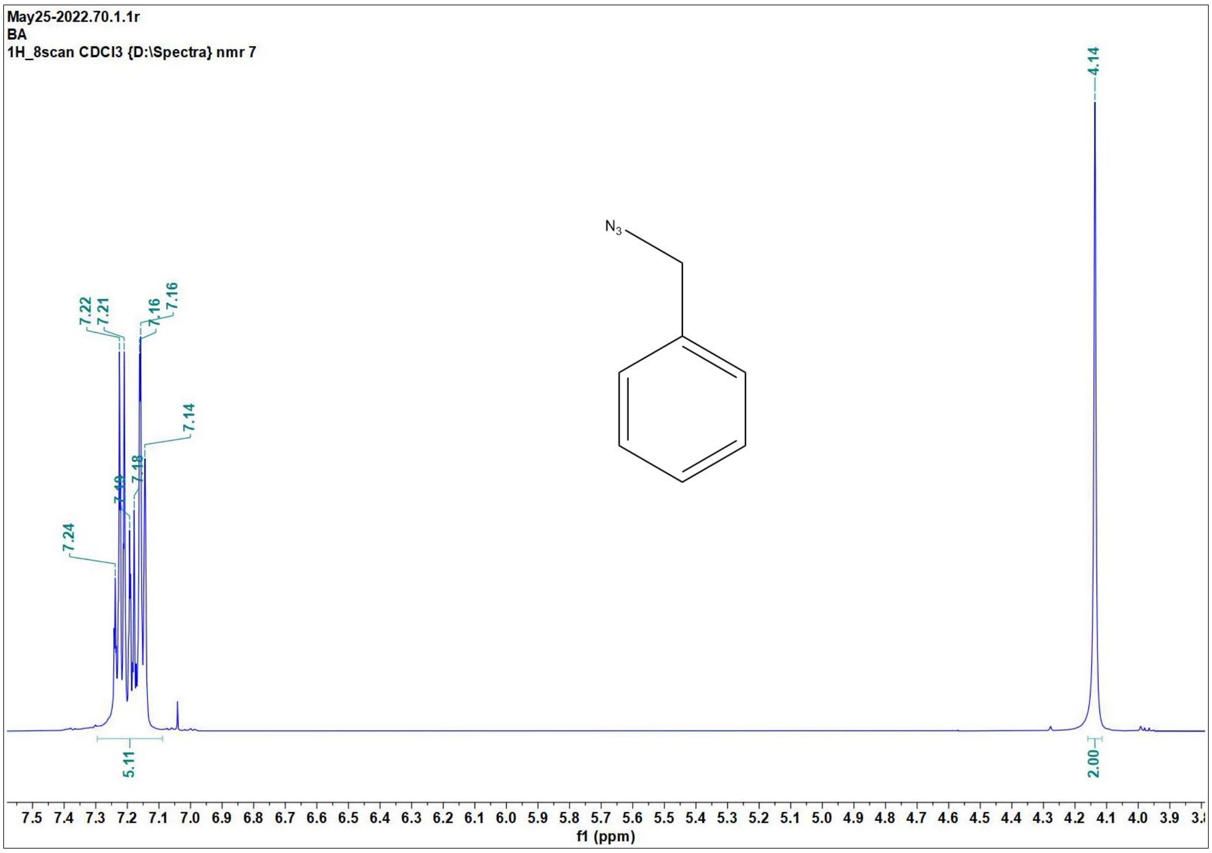

Figure S6: ^{1}H NMR spectrum of benzyl azide 5

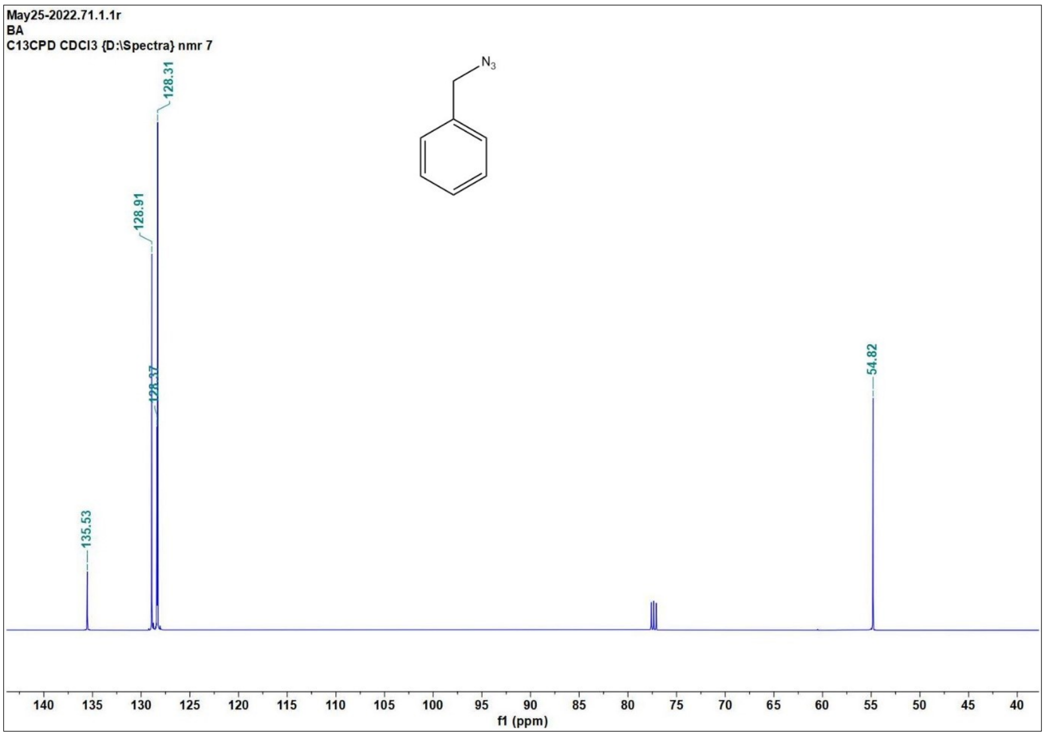

Figure S7: ^{13}C NMR spectrum of benzyl azide 5

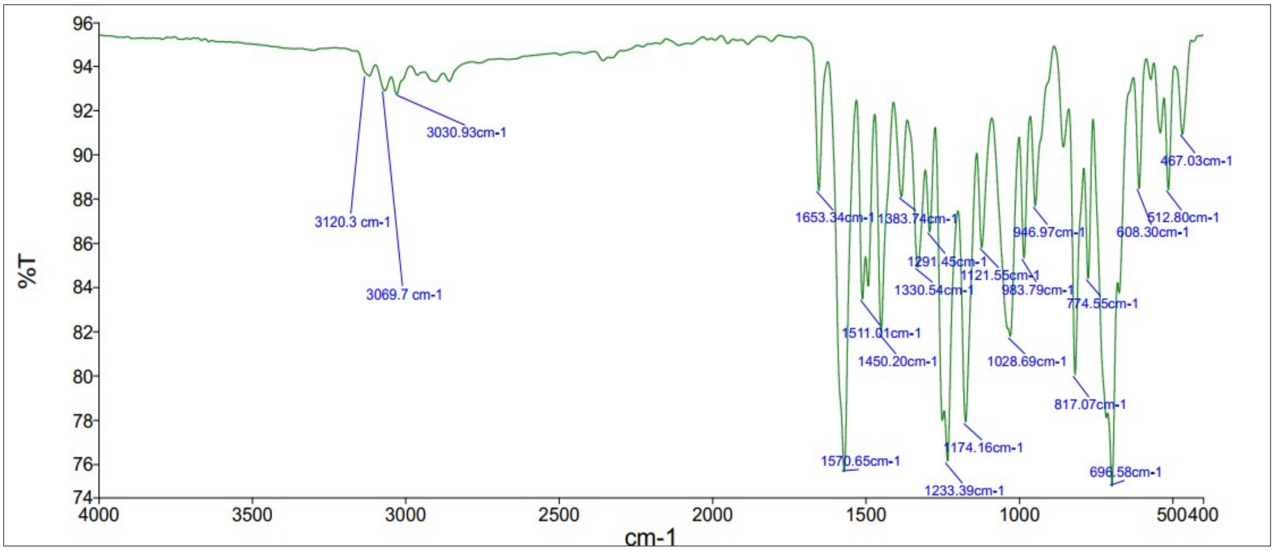

Figure S8: IR spectrum of probe CBT

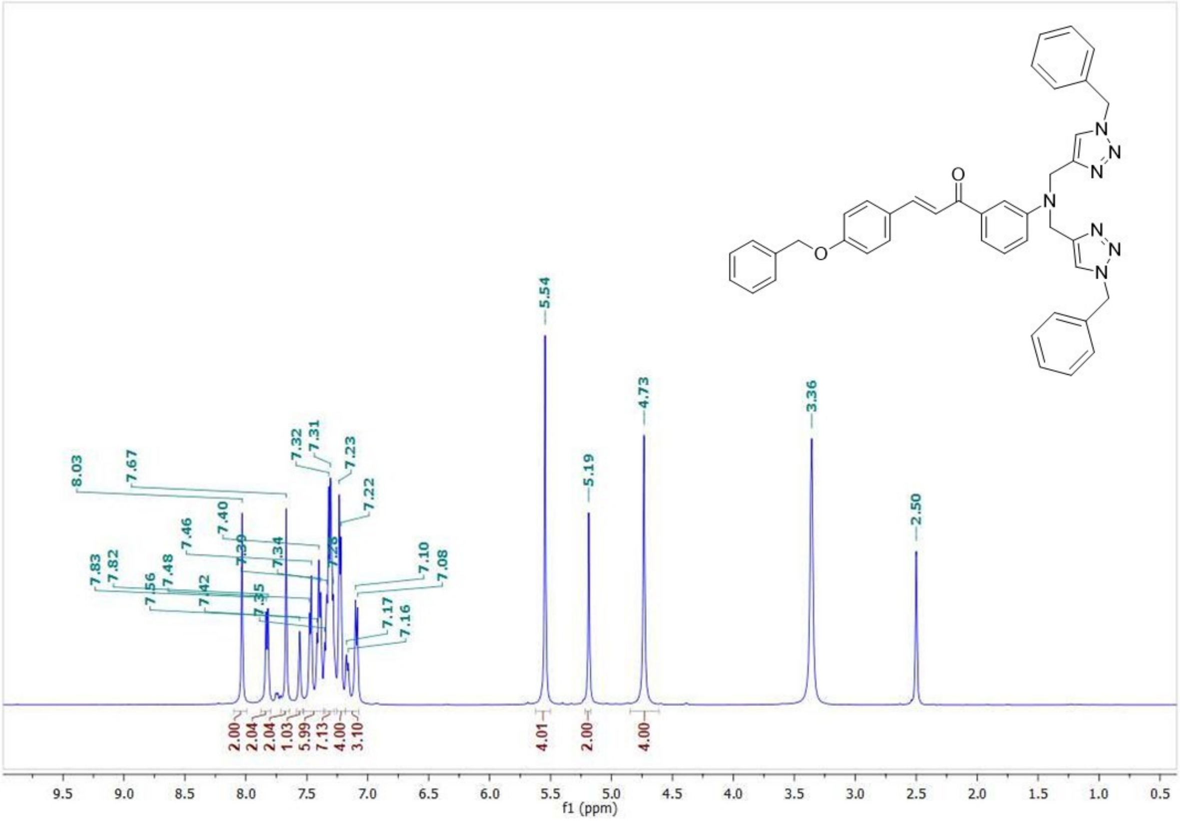

Figure S9: ^{1}H NMR spectrum of CBT

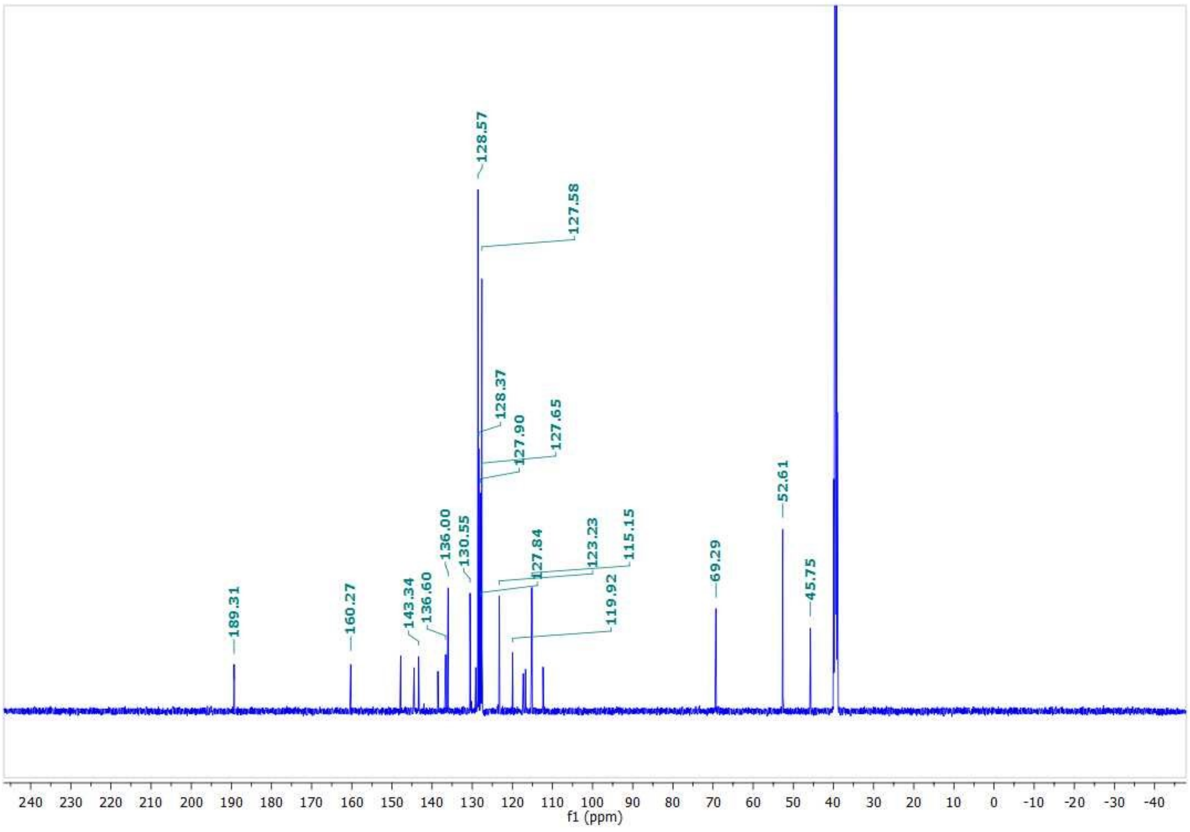

Figure S10: ^{13}C NMR spectrum of CBT

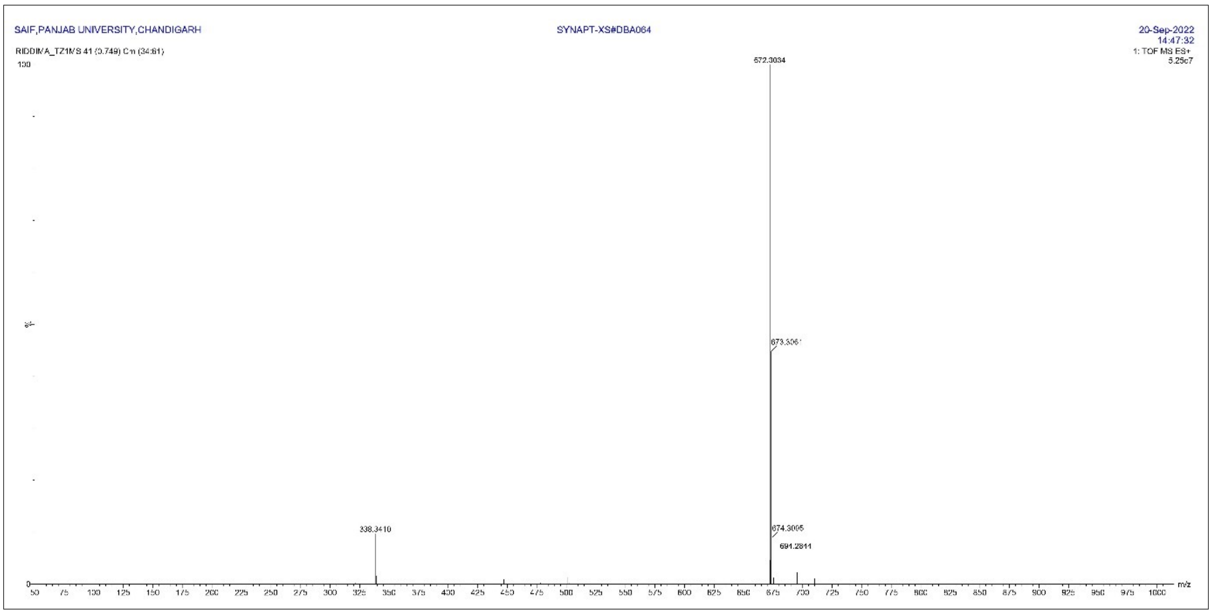

Figure S11: Mass spectrum of CBT

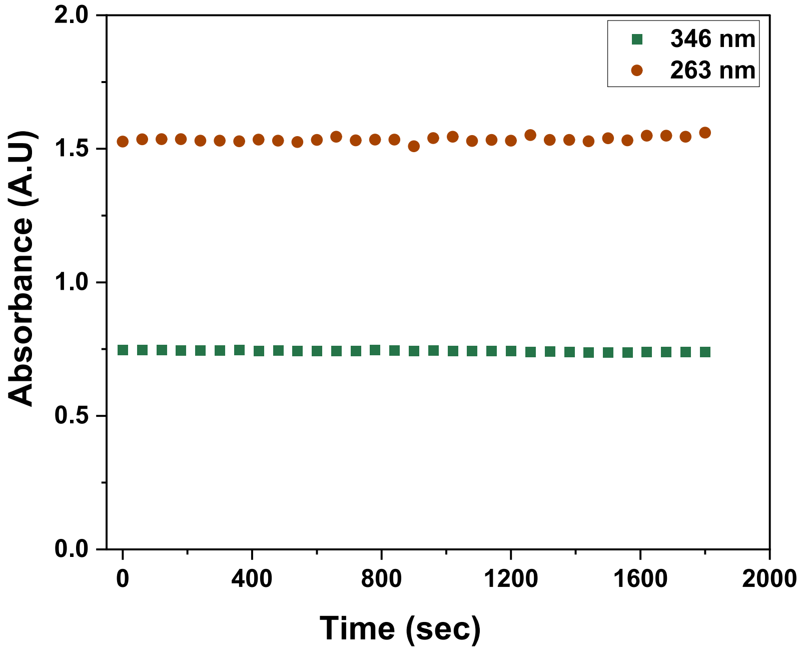

Figure S12: Time dependent spectra of CBT-Pb(II) complex displaying the trend in the absorption

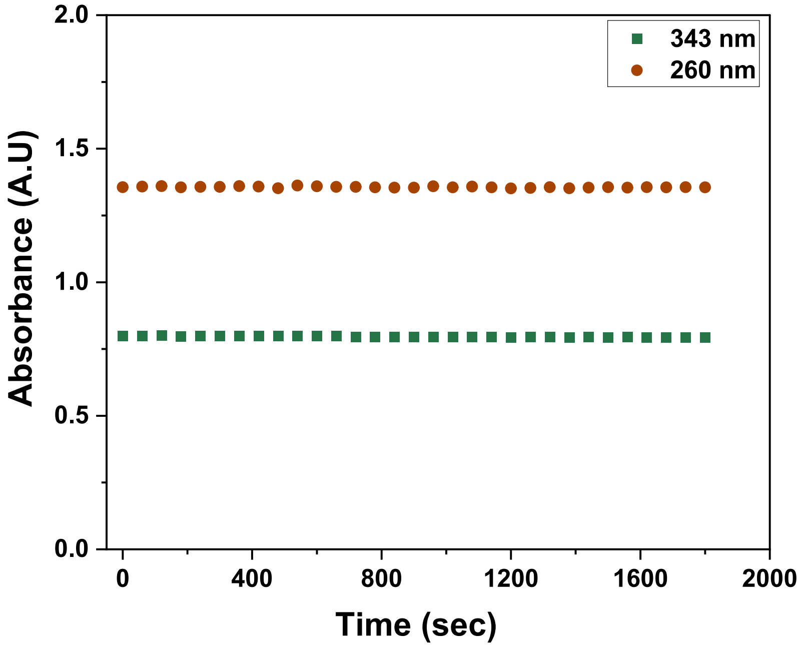

Figure S13: Time dependent spectra of CBT-Cu(II) complex displaying the trend in the absorption.

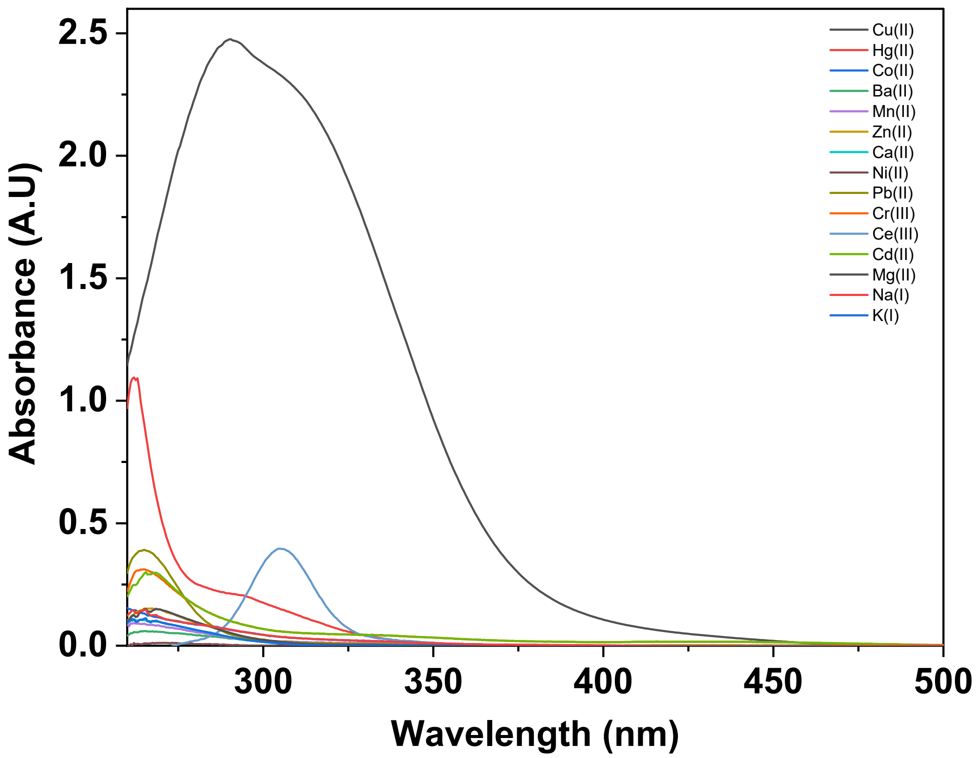

Figure S14: Individual absorption profile of all cations.

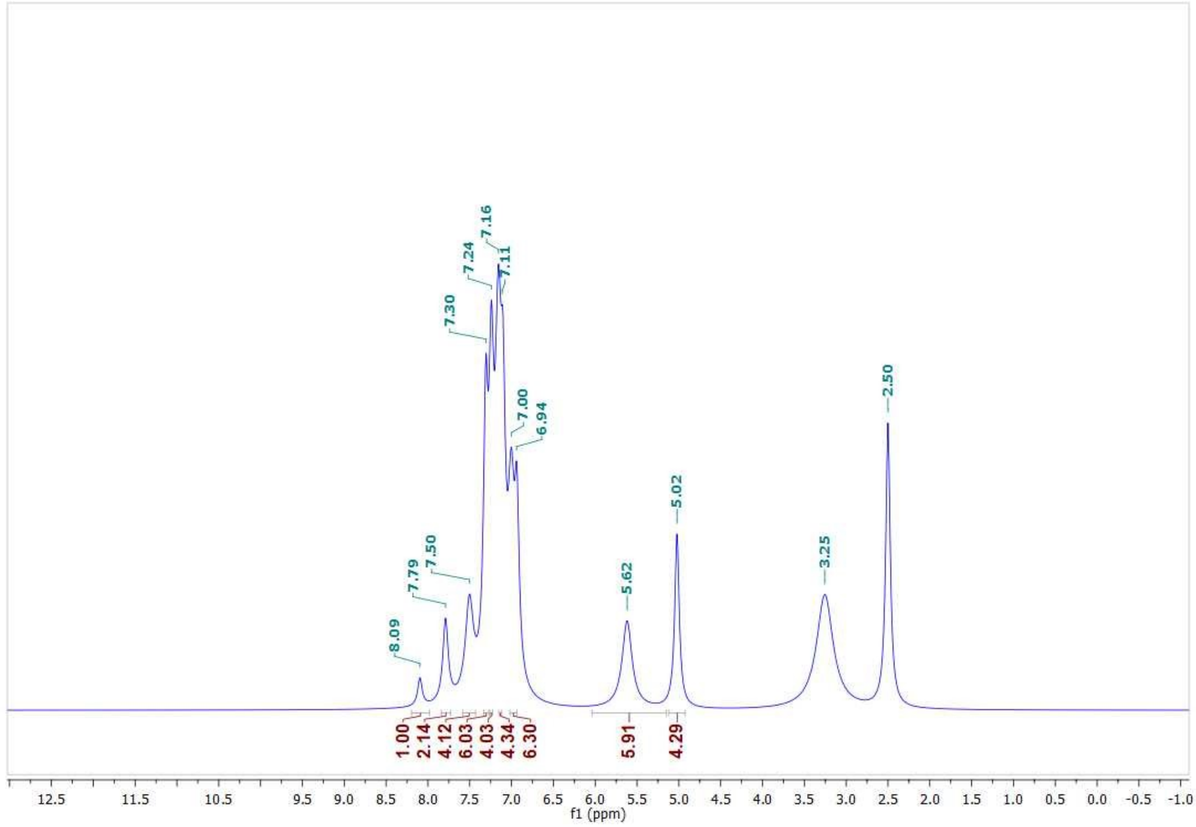

Figure S15: ^{1}H-NMR of probe CBT on interaction with the metal ion.

Table S1: Cartesian co-ordinates of CBT

Symbol	X	Y	Z
C	12.432462	-0.421049	1.816211
C	13.700227	0.15429	1.924438
C	14.526122	0.242048	0.799945
C	14.079336	-0.246207	-0.431064
C	12.810293	-0.82025	-0.535329
C	11.975678	-0.912067	0.585965
C	8.349002	-0.764365	0.036491
C	7.81399	-2.055988	0.128935
C	6.441198	-2.242687	-0.046094
C	5.569985	-1.173637	-0.315488
C	6.136838	0.119306	-0.401562
C	7.495073	0.323221	-0.230323
C	4.147834	-1.44746	-0.488124
C	3.159884	-0.572705	-0.781693

C	1.757578	-1.035272	-0.900162
C	0.698447	-0.049026	-1.310893
O	1.448908	-2.20486	-0.65557
C	-0.636327	-0.454517	-1.165784
C	-1.708419	0.382583	-1.526922
C	-1.390656	1.647036	-2.080903
C	-0.065247	2.039778	-2.241743
C	0.989555	1.211339	-1.853666
N	-3.045199	0.003873	-1.351071
C	-4.061993	0.607187	-2.215555
C	-4.659441	1.881045	-1.678004
N	-5.131278	2.851489	-2.51417
N	-5.63279	3.827864	-1.802523
N	-5.487267	3.493062	-0.500964
C	-4.879083	2.286354	-0.376843
C	-5.921699	4.420387	0.544494
C	-4.77804	5.17339	1.197972
C	-3.974848	6.038292	0.440044
C	-2.931117	6.74056	1.043258
C	-2.680392	6.590913	2.411821
C	-3.477087	5.733272	3.172298
C	-4.520633	5.026801	2.565547
O	9.66686	-0.453541	0.186966
C	-3.335071	-1.307983	-0.767599
C	-4.708622	-1.425796	-0.174862
N	-5.091907	-0.727618	0.937481
N	-6.309881	-1.064316	1.262313
N	-6.731543	-1.98702	0.369021
C	-5.759889	-2.241833	-0.543638
C	-8.067387	-2.573143	0.4953
C	-8.041944	-4.061852	0.783416
C	-8.708604	-4.956985	-0.060647

C	-8.707174	-6.328578	0.214074
C	-8.031901	-6.814755	1.334741
C	-7.36151	-5.925193	2.181933
C	-7.368319	-4.557027	1.909891
C	10.599845	-1.512777	0.46728
H	11.790793	-0.487936	2.691181
H	14.043846	0.530349	2.883707
H	15.51398	0.68588	0.883392
H	14.718043	-0.182148	-1.307209
H	12.463507	-1.198159	-1.493826
H	8.444384	-2.912759	0.333716
H	6.034704	-3.247957	0.027856
H	5.501992	0.976365	-0.602891
H	7.927889	1.316134	-0.295877
H	3.844602	-2.486552	-0.36556
H	3.370524	0.481437	-0.918401
H	-0.791144	-1.453526	-0.781259
H	-2.178102	2.333375	-2.367243
H	0.144406	3.015064	-2.671895
H	2.009944	1.54327	-2.002583
H	-4.859399	-0.128821	-2.361314
H	-3.650554	0.814383	-3.211404
H	-4.670216	1.813379	0.570041
H	-6.612494	5.107256	0.048517
H	-6.482648	3.852075	1.29169
H	-4.166682	6.152682	-0.623617
H	-2.314329	7.40672	0.446868
H	-1.86813	7.139244	2.88007
H	-3.287009	5.608983	4.234428
H	-5.136257	4.356874	3.161103
H	-2.620624	-1.476958	0.041068
H	-3.192542	-2.122558	-1.498238

H	-5.879886	-2.961817	-1.338596
H	-8.540802	-2.017049	1.308941
H	-8.627201	-2.369948	-0.422841
H	-9.234679	-4.583019	-0.935925
H	-9.229133	-7.012386	-0.448842
H	-8.026156	-7.879438	1.5487
H	-6.835528	-6.297953	3.055943
H	-6.846155	-3.868928	2.569598
H	10.310062	-2.010437	1.40169
H	10.566228	-2.249148	-0.345682
